# An Integrative Methylation‐Metabolism Gene Signature Defines Prognosis and Immunosuppressive Microenvironment in Prostate Cancer

**DOI:** 10.1111/cbdd.70360

**Published:** 2026-07-20

**Authors:** Chao Zhang, Likun Liu, Kai Wu

**Affiliations:** ^1^ Department of Urology Shanxi Hospital Affiliated to Cancer Hospital, Chinese Academy of Medical Sciences/Cancer Hospital Affiliated to Shanxi Medical University Taiyuan China; ^2^ Department of Oncology Shanxi Traditional Chinese Medical Hospital Taiyuan China

**Keywords:** amino acid metabolism, biomarker validation, DNA methylation, immunotherapy, prognostic signature, prostate cancer, tumor microenvironment

## Abstract

The synergistic crosstalk between epigenetic dysregulation and metabolic reprogramming underlies to prostate cancer (PCa) development and treatment resistance, yet an integrated prognostic signature reflecting this nexus remains poorly defined. We developed and validated a gene signature associated with methylation and amino acid metabolism for patient stratification and exploring its connection to tumor microenvironment (TME) remodeling. RNA sequencing data and independent datasets were integrated with predefined gene sets for DNA methylation (*n* = 79) and amino acid metabolism (*n* = 471). A analytical workflow was employed: identification of hub genes and least absolute shrinkage and selection operator (LASSO)—Cox modeling; construction of a prognostic nomogram; comprehensive TME profiling; and validation through single‐cell RNA sequencing (scRNA‐seq) cellular dynamics analysis and immunohistochemistry (IHC) on a prostate cancer tissue microarray. A novel six‐gene prognostic model (ASPM, WDR86, CCK, HOXA2, EGF, ZFHX4) was developed. This model efficiently discriminates patients into groups based on risk level though divergent overall survival (*p* < 0.001) and exhibited high predictive accuracy in external validation sets (3‐year area under the curve (AUC) = 0.87). A nomogram incorporating the signature, pathologic T stage, and Gleason score surpassed individual clinical factors (5‐year AUC = 0.73). Functional annotation indicated that high‐risk tumors were characterized by downregulated androgen response and activated E2F/G2M checkpoint pathways. The signature was correlated with an immunosuppressive TME, which was supported by a negative correlation between ZFHX4 and monocyte infiltration (*r* = −0.37, *p* < 0.001) and a positive correlation between ASPM and activated CD4^+^T cells (*r* = 0.44, *p* < 0.001). Single‐cell trajectory analysis exhibited that epithelial cells, fibroblasts, and natural killer T (NKT) cells was key cellular expressors of the signature. We utilized immunohistochemistry (IHC) and quantitative real‐time polymerase chain reaction (qRT‐PCR) to confirm the differential expression. We developed and validated an integrative methylation‐amino acid metabolism gene signature that effectively predicts prognosis and reflects an immunosuppressive TME in PCa. This study provides a translational framework for precision oncology, bridging epigenetic‐metabolic crosstalk to disease aggressiveness, and offers potential biomarkers for informing risk‐stratified therapy and immunotherapy approaches.

## Graphical abstract

This study developed a 6‐gene prognostic model integrating methylation and amino acid metabolism to predict PCa outcomes and reveal immunosuppressive TME.

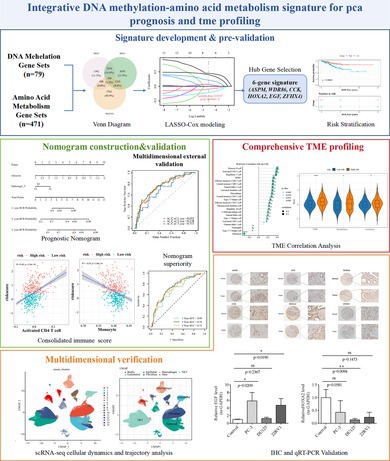

## 1 Introduction

Prostate carcinoma (PCa) represents a malignancy with high incidence rates and a predominant source of cancer‐associated mortality in the male population globally (Bray et al. 2024; Siegel et al. 2023; Kour et al. 2023). While patients with localized disease often have favorable outcomes with primary local treatments such as radical prostatectomy or radiotherapy, treating advanced and metastatic castration‐resistant prostate cancer (mCRPC) continues to be a substantial clinical problem (Eeles et al. 2014; Litwin and Tan 2017). The current treatment for this malignancy is centered on androgen deprivation therapy (ADT), chemotherapy, and next‐generation hormonal agents (Lu et al. 2023). However, their long‐term efficacy is limited by acquired resistance and cumulative toxicities (Watson et al. 2015; Cornford et al. 2021). Key biomarkers that could refine prognosis, guide risk‐based approaches, and reveal actionable therapeutic vulnerabilities are urgently required.

Tumorigenesis and progression are driven by the acquisition of multiple distinctive hallmarks (Prado et al. 2023). Among these, epigenetic alterations and metabolic rewiring are now recognized as core drivers (Hanahan and Weinberg 2011; Pavlova and Thompson 2016). DNA methylation regulates gene expression patterns fundamental to cellular function and fate (Dong, Jin, et al. 2023). This dysregulation involves genome‐wide DNA hypomethylation and promoter‐selective DNA hypermethylation of tumor suppressors (Jones 2012; You and Jones 2012). Beyond these canonical roles, amino acids collectively shape the epigenetic landscape through one‐carbon metabolism. Serine serves as the predominant one‐carbon donor: serine hydroxymethyltransferase 1/2 (SHMT1/2) transfers a one‐carbon unit into the folate cycle during serine‐to‐glycine conversion, ultimately replenishing the S‐adenosylmethionine (SAM) pool available for DNA methyltransferase (DNMT) activity (Ducker and Rabinowitz 2017; Cuyàs et al. 2018). Glutamine exerts a dual influence: its catabolism generates α‐ketoglutarate (α‐KG), the obligate co‐substrate of ten‐eleven translocation (TET) dioxygenases that mediate active DNA demethylation, while simultaneously competing for one‐carbon intermediates through nucleotide biosynthesis (Altman et al. 2016; Carey et al. 2015). Arginine‐derived polyamines further modulate chromatin compaction and cytosine‐phosphate‐guanine (CpG) site accessibility to both DNMTs and TET enzymes (Casero Jr et al. 2018). Importantly, the methylation state of any genomic locus represents a dynamic equilibrium between DNMT‐driven methylation (SAM‐dependent) and TET‐driven demethylation (α‐KG‐dependent) (Wu and Zhang 2017; Lienert et al. 2011). Dysregulation of tricarboxylic acid (TCA) cycle flux leads to succinate and fumarate accumulation, which competitively inhibits α‐KG‐dependent dioxygenases and enforces hypermethylation even when α‐KG production is not limiting (Xu et al. 2011)—explaining how heightened glutaminolysis can paradoxically coexist with progressive tumor suppressor silencing. This bidirectional metabolic–epigenetic interplay, whereby metabolites drive chromatin modifications and epigenetic regulators in turn reshape metabolic enzyme expression, creates feed‐forward loops implicated in treatment resistance and poor outcomes across malignancies.

In PCa, the mechanistic links between amino acid metabolism and methylation reprogramming are increasingly well‐documented. The near‐universal hypermethylation and transcriptional silencing of glutathione S‐transferase pi 1 (GSTP1)—one of the earliest and most consistent epigenetic events in PCa—has been mechanistically linked to enhanced methionine cycle activity that elevates SAM levels, driving DNMT3a/3b‐mediated promoter methylation (Yegnasubramanian et al. 2004). In castration‐resistant PCa (CRPC), glutaminase 1/2 (GLS1/GLS2)‐driven glutaminolysis is accompanied by TCA metabolite imbalance and consequent TET2 functional suppression, contributing to the progressive genome‐wide hypermethylation associated with disease progression (Eidelman et al. 2017). Furthermore, a reciprocal regulatory relationship exists between androgen receptor (AR) signaling and serine–glycine one‐carbon flux: AR signaling modulates the expression of one‐carbon metabolic enzymes, while the resulting shifts in SAM availability reconfigure the methylation landscape that governs AR pathway activity, establishing a feed‐forward loop that drives castration resistance (Mentch and Locasale 2016). However, despite growing recognition of this interplay, existing integrative studies in PCa have addressed only partial aspects of this axis. Xu et al. constructed a multi‐omics prognostic model integrating DNA methylation with transcriptomic profiles, yet focused exclusively on the epigenetic layer without incorporating amino acid metabolic reprogramming as a co‐regulatory dimension (Xu et al. 2023). Similarly, Strmiska et al. systematically characterized amino acid metabolic alterations in PCa but did not extend their analysis to downstream epigenetic consequences, nor did they yield a clinically applicable prognostic signature. Critically, no existing study has simultaneously integrated methylation‐associated and amino acid metabolism‐related gene programs to construct a unified prognostic model, nor has any prior work systematically characterized how this combined axis shapes the tumor immune microenvironment in PCa.

To bridge this gap, we conducted an integrative multi‐omics analysis based on the hypothesis that aberrant methylation–amino acid metabolic crosstalk promotes immune evasion in PCa—specifically, by shifting the SAM/α‐KG ratio toward promoter hypermethylation of immune‐activating genes and by generating metabolic byproducts such as polyamines and succinate that impair CD8^+^ T cell function and facilitate recruitment of immunosuppressive populations including regulatory T cells (Tregs) and M2‐like macrophages. We first conducted a systematic bioinformatics evaluation to assess the outcome‐associated significance of this axis. We established a risk assessment system and a nomogram‐based tool. We characterized the distinct biological pathways, immune contexture, and cellular states associated with high‐risk disease. Finally, validation in prostate cancer tissues and cell lines confirmed the protein expression and prognostic significance of the signature genes. This work aims to establish a novel methylation‐metabolism framework for prognosis prediction and to reveal its underlying biology in PCa.

## 2 Materials and Methods

### 2.1 Data Preprocessing

Transcriptome sequencing data together with matched patient characteristics annotations for prostate adenocarcinoma (PRAD) cohort were retrieved through The Cancer Genome Atlas (TCGA) portal, comprising 499 primary tumor specimens and 52 adjacent normal ones, which served as training set. For independent validation, the gene expression dataset GSE70769 (Platform: GPL10558), containing 92 tumor samples with clinical information, was acquired. Single‐cell transcriptomics from the GSE181294 series (Platform: GPL18573), which includes 26 tumor and 25 normal prostate samples, was acquired from Gene Expression Omnibus (GEO) for cellular heterogeneity analysis. The MRG panel was constructed by integrating two complementary sources. First, curated methylation‐regulatory gene sets were retrieved from MSigDB C2 (curated gene sets) and C5 (ontology gene sets) collections, specifically targeting pathways related to “DNA methylation,” “epigenetic regulation,” and “chromatin modification.” Second, core DNA methylation regulators—including DNA methyltransferases (DNMTs), ten‐eleven translocation (TET) dioxygenases, and methyl‐CpG‐binding domain (MBD) proteins—were supplemented based on experimentally validated entries reported in references (Nie et al. 2022; He et al. 2022; Wu et al. 2023; Dong et al. 2022). Inclusion criteria were: (1) protein‐coding genes only; (2) genes with stable expression across tumor samples (coefficient of variation < 1.5) and clear functional annotation in the context of DNA methylation regulation; (3) removal of duplicates and genes with negligible expression. The final MRG panel comprised 79 genes. The AAMRG panel was curated through a systematic multi‐database integration strategy. Amino acid metabolism‐related gene sets were retrieved from four authoritative resources: (1) KEGG Pathway database (amino acid biosynthesis and degradation pathways); (2) Gene Ontology (GO) terms annotated under “amino acid metabolic process” (GO:0006520) and related child terms; (3) MSigDB Hallmark gene set “HALLMARK_AMINO_ACID_METABOLISM” (v7.5); and (4) Reactome pathways under the “Amino acid and derivative metabolism” hierarchy. Retrieved gene lists were merged and deduplicated. Subsequently, non‐protein‐coding genes and genes with low expression (mean normalized count < 1 across the TCGA‐PRAD cohort) were excluded. This process yielded a final curated panel of 471 AAMRGs. Additionally, promoter‐region DNA methylation beta values for the six prognostic genes were obtained from the TCGA‐PRAD 450 K/850 K Illumina methylation array platform for epigenetic characterization. CpG probes annotated to TSS200 and TSS1500 regions were selected to represent promoter‐region methylation. Differential methylation between tumor and matched adjacent normal tissues was assessed using the Wilcoxon rank‐sum test. Pearson correlation analysis was performed to examine associations between promoter methylation beta values and composite risk scores. Patients were stratified into high‐ and low‐methylation groups using the median beta value as the cutoff, and Kaplan–Meier survival analysis with log‐rank testing was conducted to evaluate prognostic associations. To externally validate the six‐gene signature, an independent dataset GSE116918 (GPL25318) was obtained from GEO. The locked risk score formula derived from TCGA‐PRAD was directly applied without modification. Patients were stratified into high‐ and low‐risk groups using the same cut‐off as in the training set. Kaplan–Meier analysis, time‐dependent ROC, risk score distribution, and expression heatmap were used to evaluate prognostic performance and consistency.

### 2.2 Profiling of Differentially Regulated Transcripts

We performed differential transcriptional profiles (tumor versus normal adjacent tissues) utilizing DESeq2‐R‐package (v1.36.0) (Love et al. 2014). We obtained differentially expressed genes (DEGs1). To profile pathway activity, enrichment levels were calculated at the individual sample level (v1.42.0) using single‐sample gene set enrichment analysis (ssGSEA) (Hänzelmann et al. 2013). Samples were stratified into high versus low AAMRG activity subsets using the median ssGSEA score as the cutoff, a widely adopted approach for continuous biomarker dichotomization in transcriptomic prognostic studies. To verify the robustness of this stratification strategy, sensitivity analyses were performed using two alternative grouping methods: tertile‐based stratification and the maximally selected rank statistics method (optimal cutpoint). We conducted prognostic analysis to compare overall survival (OS) between these groups. Subsequently, differential expression analysis was performed between these high and low activity groups using the same statistical thresholds to identify DEGs2. Multivariate Cox proportional hazards regression was subsequently performed to assess the independent prognostic value of the AAMRG score after adjustment for age, Gleason score, pathological T stage, and PSA level. Results are summarized as hazard ratios (HR) with 95% confidence intervals (CI) and two‐tailed *p*‐values.

### 2.3 Network‐Based Analysis of Co‐Expressed Genes

An MRG activity score was similarly calculated for each sample using ssGSEA. Utilizing the weighted gene co‐expression network analysis (WGCNA) package (v1.71) (Langfelder and Horvath 2008), gene modules were identified, and their module eigengenes (MEs) were calculated. Pearson correlation between each ME and the continuous MRG ssGSEA score was assessed. To objectively select key modules, a quantitative criterion was applied: modules with an absolute module‐trait correlation |*r*| > 0.4 and *p* < 1 × 10^−20^ were considered statistically eligible. Applying this criterion, three modules qualified: MEgreen (*r* = −0.565, *p* = 5.2 × 10^−42^), MEblue (*r* = −0.411, *p* = 5.7 × 10^−21^), and MEturquoise (*r* = 0.407, *p* = 9.29 × 10^−21^). To maintain analytical focus and maximize biological interpretability, we selected one module per directional extreme: MEgreen, representing the strongest negative correlation, and MEturquoise, representing the strongest positive correlation with the MRG score. MEblue, while statistically eligible, exhibited a correlation magnitude comparable to MEturquoise (|*r*| = 0.411 vs. 0.407) and shared the same directional trend as MEgreen; its inclusion would have introduced redundancy without substantially altering the gene pool for downstream analysis. The constituent genes of MEgreen and MEturquoise were therefore extracted as key module genes for subsequent analyses.

### 2.4 Selection and Functional Enrichment of Candidate Genes

Selected biomarkers were defined as the overlap between DEGs1 and DEGs2, and core genes based on network analysis of WGCNA. Gene Ontology (GO) and Kyoto Encyclopedia of Genes and Genomes (KEGG) pathway enrichment analyses were performed on this candidate gene set (Wu et al. 2021).

### 2.5 Construction and Validation of a Prognostic Signature

Using OS data from 493 TCGA‐PRAD samples with complete follow‐up, We selected transcripts with substantial predictive relevance (*p* < 0.05) for further analysis. To prevent overfitting and select the most robust predictors, we used the cv.glmnet function in the glmnet package to perform the least absolute shrinkage and selection operator (LASSO) Cox regression with 10‐fold cross‐validation. The optimal penalty parameter *λ* was determined by the minimal cross‐validation error (*λ*.min). To ensure that the results were not dependent on a single data partition, repeated cross‐validation was further conducted to assess the stability of both the model structure and the selected coefficients, confirming that the six‐gene signature was consistently identified across iterations. A risk score for each patient was calculated using the formula: Risk Score = Σ (Expression of Gene*ᵢ* × LASSO Coefficient*ᵢ*). Patients were stratified into high‐ and low‐risk groups using the optimal cut‐off value determined by the surv_cutpoint() function in the survminer package, which identifies the threshold that maximizes the log‐rank test statistic. To evaluate prognostic performance and account for potential optimism in training‐set estimates, time‐dependent AUC was assessed in the TCGA cohort using the same 10‐fold cross‐validation framework. In each fold, the model was trained on 90% of samples and tested on the held‐out 10%; AUC values were averaged across all folds to derive bias‐corrected performance estimates. The entire model‐building process was subsequently locked and applied without modification to the independent GSE70769 validation cohort. To further interrogate the prognostic utility of the model across complementary clinical endpoints, biochemical recurrence‐free survival (BCR‐FS) was additionally evaluated within the GSE70769 cohort. The locked risk score formula was applied without modification. Risk score distribution, recurrence event status, time‐dependent ROC analysis, and Kaplan–Meier analysis of BCR‐FS were performed using identical parameters as described above.

### 2.6 Independent Prognostic Assessment and Nomogram Development

Cox regression analyses were performed to investigate the independent prognostic value of the risk score alongside clinical characteristics including age, pathologic T stage, N stage, Gleason score, and prostate‐specific antigen (PSA) level. Prior to model construction, the proportional hazards (PH) assumption was formally assessed for all variables using the Schoenfeld residuals test. PSA was found to significantly violate the PH assumption (Schoenfeld residual test *p* < 0.05) and was therefore excluded from the multivariable Cox model. All remaining variables satisfied the PH assumption (Schoenfeld residual test *p* > 0.05) and were retained for further analysis. Pathological T stage was treated as an unordered nominal categorical variable with T2 as the reference level, and dummy variables were created for T3 and T4 stages. Gleason score was categorized into four clinically established groups (≤ 6, 7, 8, and ≥ 9) and modeled as an ordinal variable; a parallel analysis using unordered dummy coding yielded consistent results, confirming model robustness. Ties in survival time were handled using the Efron method, which is the standard approach in Cox proportional hazards regression. Informed by the results of the multivariate Cox model, a nomogram was constructed to predict 1‐, 3‐, and 5‐year OS probabilities. The nomogram's predictive capability was validated by calibration plots (with 1000 bootstrap resamples) and decision curve analysis (DCA).

### 2.7 Functional and Pathway Evaluations

Gene Set Enrichment Analysis (GSEA) and the top three enriched gene datasets were conducted. To further compare biological activities between risk‐based groups, Gene Set Variation Analysis (GSVA) was performed. Differential pathway activity was assessed using the limma package.

### 2.8 Tumor Microenvironment and Immunotherapy Response Assessment

The relative abundance of 28 immune cell types in each sample was quantified by calculating their ssGSEA scores. Variations in immunological cellular penetration and manifestation of 13 immunological checkpoint genes between risk categories were compared using the Wilcoxon test. Correlation analyses (Pearson or Spearman) were performed among prognostic genes, risk scores, and immune features. We utilized the ESTIMATE algorithm to estimate to calculateImmuneScore, StromalScore, and ESTIMATEScore together with tumor purity. We conducted TIDE score to assess immunotherapy responsiveness (Tumor Immune Dysfunction and Exclusion).

### 2.9 Drug Sensitivity Prediction and Molecular Docking

50% inhibitory concentration (IC50) of 138 anti‐cancer compounds was predicted for each sample (Xiao et al. 2022; Jiang et al. 2022). Drugs with notably different IC50 values across risk categories were identified. For the top two candidates, molecular docking was performed. Protein structures of the prognostic genes were retrieved from the AlphaFold Database or RCSB PDB, and ligand structures were downloaded from PubChem. Docking was conducted using AutoDock Vina, and results were visualized with PyMOL (v3.11.2) (Rosignoli and Paiardini 2022). A regulatory network of microRNAs (miRNAs) and transcription factors (TFs) targeting the prognostic genes was predicted (Shannon et al. 2003).

### 2.10 Single‐Cell Transcriptomic Data Processing

The single‐cell RNA sequencing dataset (GSE181294) was conducted utilizing the Seurat pipeline (v3.1.5) (Tan et al. 2023). Quality control was performed independently for each sample to account for inter‐sample variability. Cells were retained if they met all of the following criteria: unique feature counts between 200 and 6000; UMI counts > 500; and mitochondrial gene percentage < 20%. Doublets and low‐quality libraries were additionally removed using standard procedures. These thresholds were applied uniformly across all 26 tumor and 25 normal samples to ensure data consistency. Data normalization, scaling, and principal component analysis (PCA) were carried out. To mitigate potential batch effects arising from the integration of multiple tumor and normal samples, batch correction was performed using the RunHarmony function (Harmony v1.0) within the Seurat pipeline, with sample identity specified as the batch covariate. Harmony‐corrected embeddings were used for all subsequent clustering, cell annotation, and trajectory analyses. Cellular subclusters were identified via graph‐based clustering on the top 50 principal components and visualized with Uniform Manifold Approximation and Projection (UMAP). Cellular annotation was performed via the SingleR algorithm (v1.8.1) (Zhang et al. 2022) and canonical marker genes. Intercellular communication analysis was performed (Fang et al. 2022; Xie et al. 2023). Key cell populations were defined by their differential expression of the prognostic signature genes. Pseudotemporal trajectory analysis was performed on the key cell populations using the Monocle2 package (v2.26.0) (Ni et al. 2023). Cells were ordered using highly variable genes identified by differential expression testing across clusters (*q* < 0.01, via the differentialGeneTest function). Dimensionality reduction was performed using the DDRTree method with reverse graph embedding to reconstruct the trajectory structure. The root state was defined based on the least differentiated cluster as determined by canonical marker gene annotation. Trajectory branching points were statistically assessed using the branch expression analysis modeling (BEAM) function to confirm the reliability of identified differentiation branches. All parameters were set to defaults and held fixed to ensure reproducibility.

### 2.11 Cell Culture

The human prostate cellular strains employed in this study comprised the non‐malignant stromal cell line WPMY‐1 and human prostate carcinoma cell lines PC‐3, DU145, and 22RV1 (sourced from Procell Life Science & Technology Co. Ltd. (Wuhan, China)). All cell lines were incubated at 37°C in a humidified atmosphere containing 5% CO_2_.Each cell line was cultured in its respective recommended medium (refer to manufacturer's protocols), containing 10% fetal bovine serum (FBS) and 1% penicillin‐streptomycin. Cell cultures were routinely monitored for Mycoplasma infection.

### 2.12 Real‐Time Quantitative Polymerase Chain Reaction (RT‐qPCR)

Abundance of prognostic gene transcripts in TCGA‐PRAD was extracted and their differential expression was analyzed to clarify their difference in normal and tumour samples. Besides, quantitative real‐time PCR (qRT‐PCR) was employed to confirm the expression of prognostic genes. For this analysis, a total of 12 cell samples were utilized for qRT‐PCR, including 3 WPMY‐1, 3 PC‐3, 3 DU145, and 3 22RV1 samples. RNA was extracted from frozen samples with TRIzol (Invitrogen, USA), quantified by NanoPhotometer N50, reverse transcribed, and subjected to qPCR. Expression levels were calculated using the 2^−ΔΔCT^ method (Primer sequences are in Table S1).

### 2.13 Immunohistochemistry and Quantitative Analysis

Tissue specimens: A prostate cancer tissue microarray (TMA; PC‐1601) containing 192 cores from 64 patients (paired tumor/adjacent normal tissues) was utilized. Antibodies and staining: IHC was performed on 4‐μm sections using the following validated primary antibodies:

- ASPM (Proteintech Group, 26223‐1‐AP, 1:100);
- CCK (Proteintech Group, 13074‐2‐AP, 1:100);
- EGF (Proteintech Group,27141‐1‐AP, 1:100);
- HOXA2 (BIOSS Biotechnology Co. Ltd., bs‐17362R, 1:200);
- WDR86 (Invitrogen, PA5‐48389, 1:25);
- ZFHX4 (BIOSS Biotechnology Co. Ltd., bs‐18523R, 1:200).

Immunohistochemical quantification was performed using the Indica Labs HALO platform. Manual delineation of the region of interest (ROI) was followed by selection of an appropriate analysis module for either positive cell counting or area quantification. The 3,3′‐diaminobenzidine (DAB) chromogen signal was defined and thresholded consistently across all slides for a given marker. The algorithm identified nuclei and expanded cytoplasmic boundaries to compute the following parameters: number of positive cells, positive area, average optical density (AOD), and total tissue area. Derived metrics included positive cell ratio (positive cells/total cells), positive cell density (positive cells/tissue area), mean optical density (MOD), positive area ratio (positive area/total tissue area), and H‐Score [∑(1 × % weak + 2 × % moderate + 3 × % strong), range 0–300]. For digital pathology, whole‐slide imaging was conducted were captured at 20× objective magnification and analyzed utilizing Aipathwell (Servicebio), an AI‐based image analysis software, which automatically quantified the above metrics via deep learning algorithms.

### 2.14 Statistical Evaluation

Every analytic calculation was performed within the R environment (v4.2.2). Cohort contrasts used Student's *t*‐test (parametric) or Wilcoxon rank‐sum test (non‐parametric). Survival differences were assessed by the log‐rank test. Correlation analyses used Pearson or Spearman methods. Differences with a two‐tailed *p*‐value < 0.05 were regarded as statistically significant.

## 3 Results

### 3.1 Acquisition of 6627 DEGs1 and 4038 DEGs2

**FIGURE 1:**
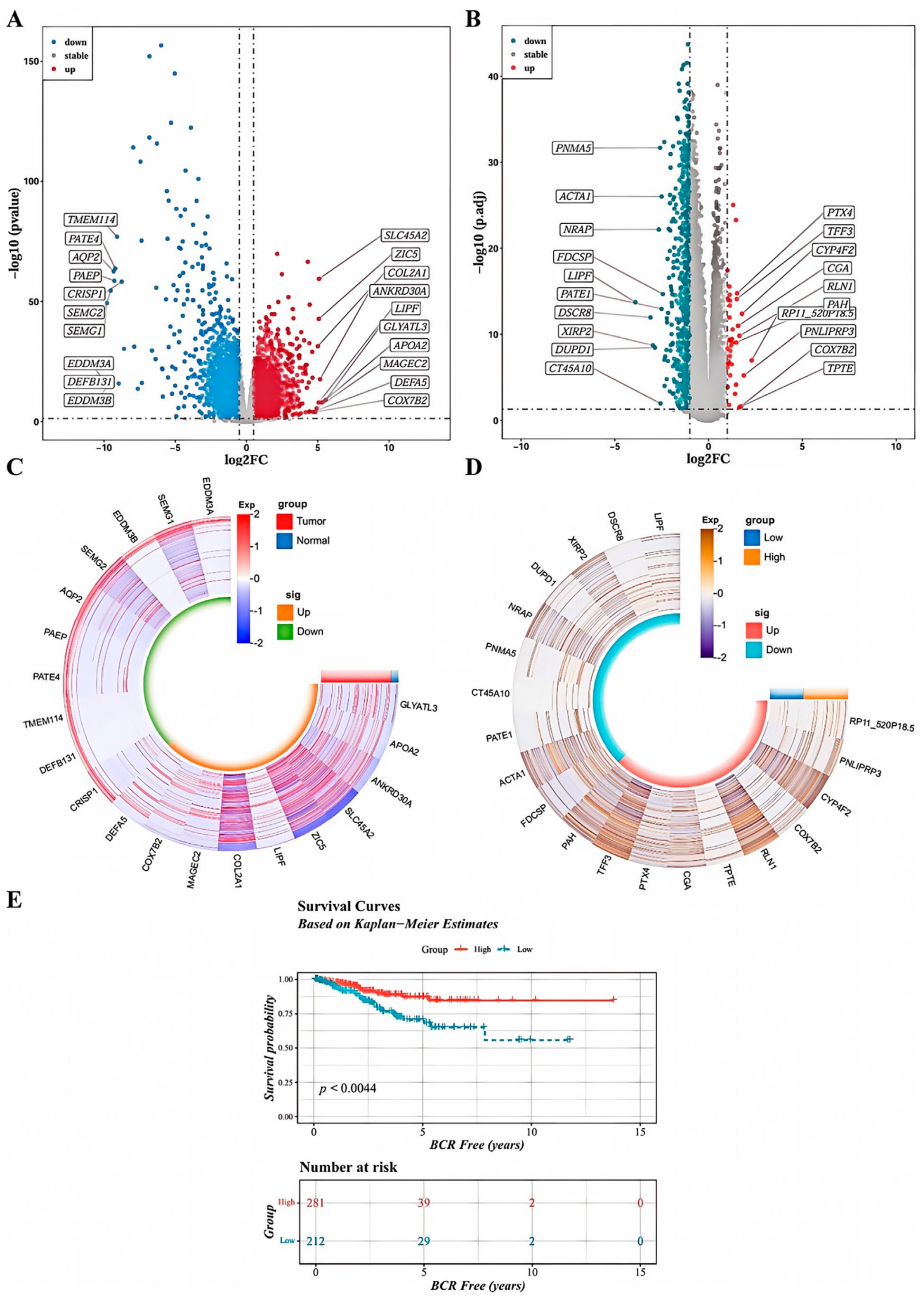
Identification of differentially expressed genes (DEGs) associated with amino acid metabolism in TCGA‐PRAD cohort. (A) Volcano plot showing DEGs between prostate cancer (PCa) tissues and adjacent normal tissues (DEGs1). Red dots: Upregulated genes (|log2FC| > 1, padj < 0.05); blue dots: Downregulated genes. (B) Volcano plot of DEGs between high and low amino acid metabolism‐related gene (AAMRG) score groups (DEGs2). High/low groups were stratified by median ssGSEA score of AAMRGs (C) Heatmap of the top 50 upregulated and downregulated DEGs1. (D) Heatmap of the top 50 DEGs2 between high and low AAMRG score groups. (E) Kaplan–Meier survival curves for patients stratified by AAMRG score (high vs. low). High AAMRG score was associated with favorable prognosis (HR = 0.42, 95% CI: 0.25–0.70, log‐rank *p* = 0.00107). Sensitivity analyses using tertile stratification (*p* = 0.00047) and optimal cutpoint method (*p* = 3.59 × 10^−5^) confirmed the robustness of this stratification.

First, a total of 6627 DEGs1 were identified in TCGA‐PRAD, 3192 of these were upregulated and 3435 were downregulated (Figure 1A,C). Significant survival differences were observed between AAMRG score groups (Figure 1E). Univariate Cox regression analysis demonstrated that a high AAMRG score was associated with favorable prognosis (HR = 0.42, 95% CI: 0.25–0.70, *p* = 0.00107). After that, differential expression analysis between high and low AAMRG score groups was performed using the same statistical thresholds as DEGs1 (|log_2_FC| > 1 and *p*_adj_ < 0.05), yielding a total of 4038 DEGs2, which included 378 overexpressed and 3660 underexpressed genes (Figure 1B,D). The complete lists of DEGs1 are provided in Tables S2 and S3, respectively. To further assess the independent prognostic value of the AAMRG score, multivariate Cox proportional hazards regression was performed adjusting for established clinical covariates including age, Gleason score, pathological T stage, and PSA level. After adjustment, the AAMRG score showed a trend toward prognostic significance (HR = 0.62, 95% CI: 0.35–1.10, *p* = 0.104), though it did not reach the threshold for statistical independence in this cohort. Pathological T3 stage (HR = 2.97, 95% CI: 1.26–7.01, *p* = 0.013) and PSA > 10 (HR = 4.08, 95% CI: 1.57–10.59, *p* = 0.0039) emerged as statistically independent prognostic factors.

### 3.2 There Were 7383 Key Module Genes in MEgreen and MEturquoise Modules

**FIGURE 2:**
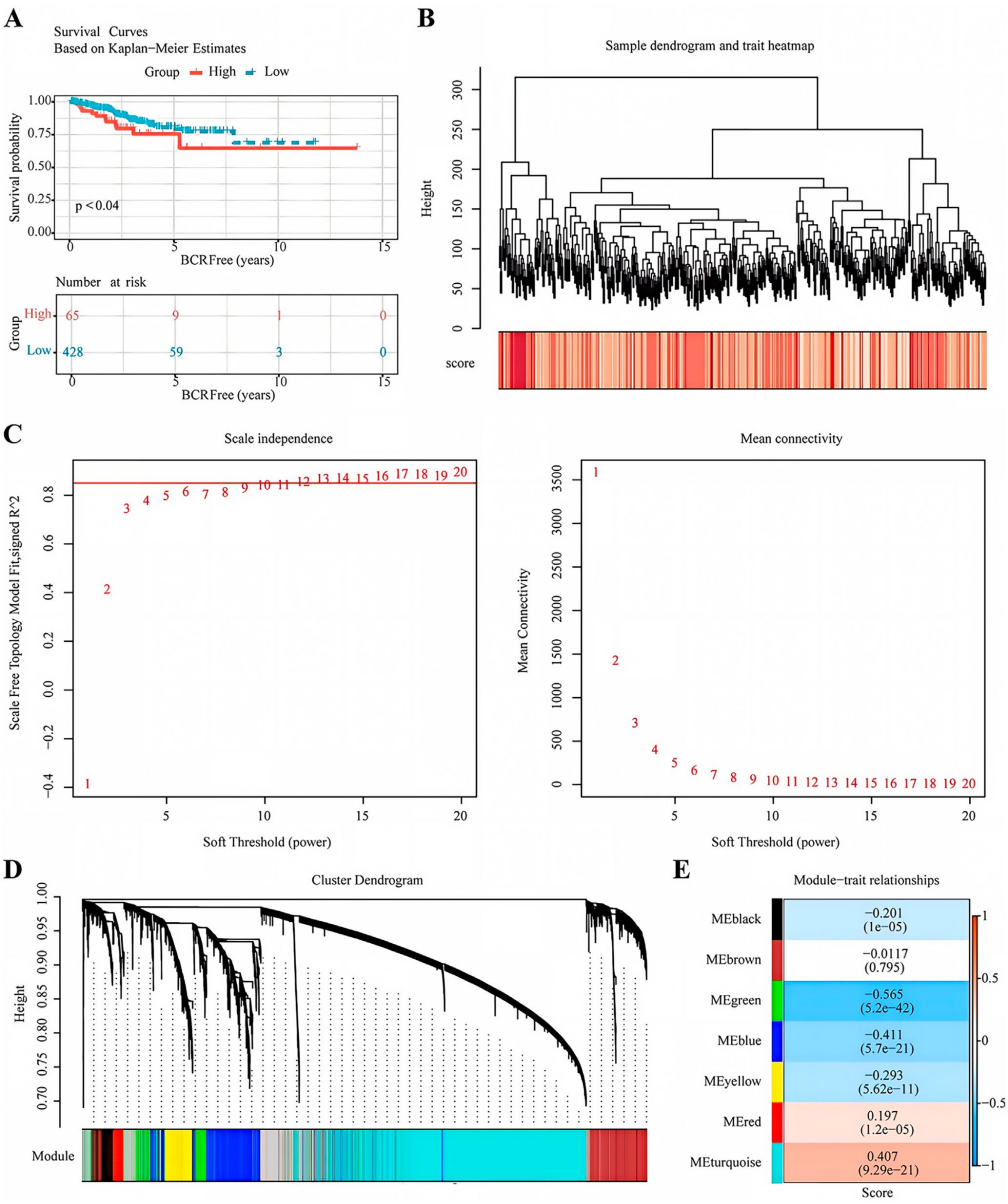
Weighted gene co‐expression network analysis (WGCNA) identifies modules correlated with methylation‐related gene (MRG) score. (A) Kaplan–Meier curves for overall survival based on MRG score groups. (B) Sample dendrogram and trait heatmap showing no outliers in TCGA‐PRAD cohort. (C) Analysis of scale‐free fit index (left) and mean connectivity (right) for various soft‐threshold powers (β). (D) Cluster dendrogram of genes, with the eight modules identified by WGCNA shown in different colors. (E) Heatmap of module‐trait relationships. Each row corresponds to a module eigengene (ME); each column to a trait (MRG score). Correlation coefficients and *p*‐values are displayed. MEgreen (negative correlation) and MEturquoise (positive correlation) were selected as key modules.

In this study, we observed a significant survival distinction in MRGs score between its high‐ and low‐risk subsets (Figure 2A). Based on this result, we performed the WGCNA analysis. Firstly, the results of cluster analysis confirmed the lack of sample outliers in TCGA‐PRAD (Figure 2B). Subsequently, identifying the optimal soft threshold to be 12, the resulting network exhibited approximate scale‐free topology (Figure 2C). After that, the tumor specimens were divided into 8 gene modules (Figure 2D), among which MEgreen (Cor = −0.565, *p* value = 7.42 × 10^42^), MEturquoise (Cor = 0.407, *p* value = 3.98 × 10^−21^) presented the largest negative and positive correlation with MRGs score, respectively, which contained 7383 genes that were used as key module genes for subsequent analyses (Figure 2E).

### 3.3 The 304 Candidate Genes Were Identified for Functional Enrichment Analysis

**FIGURE 3:**
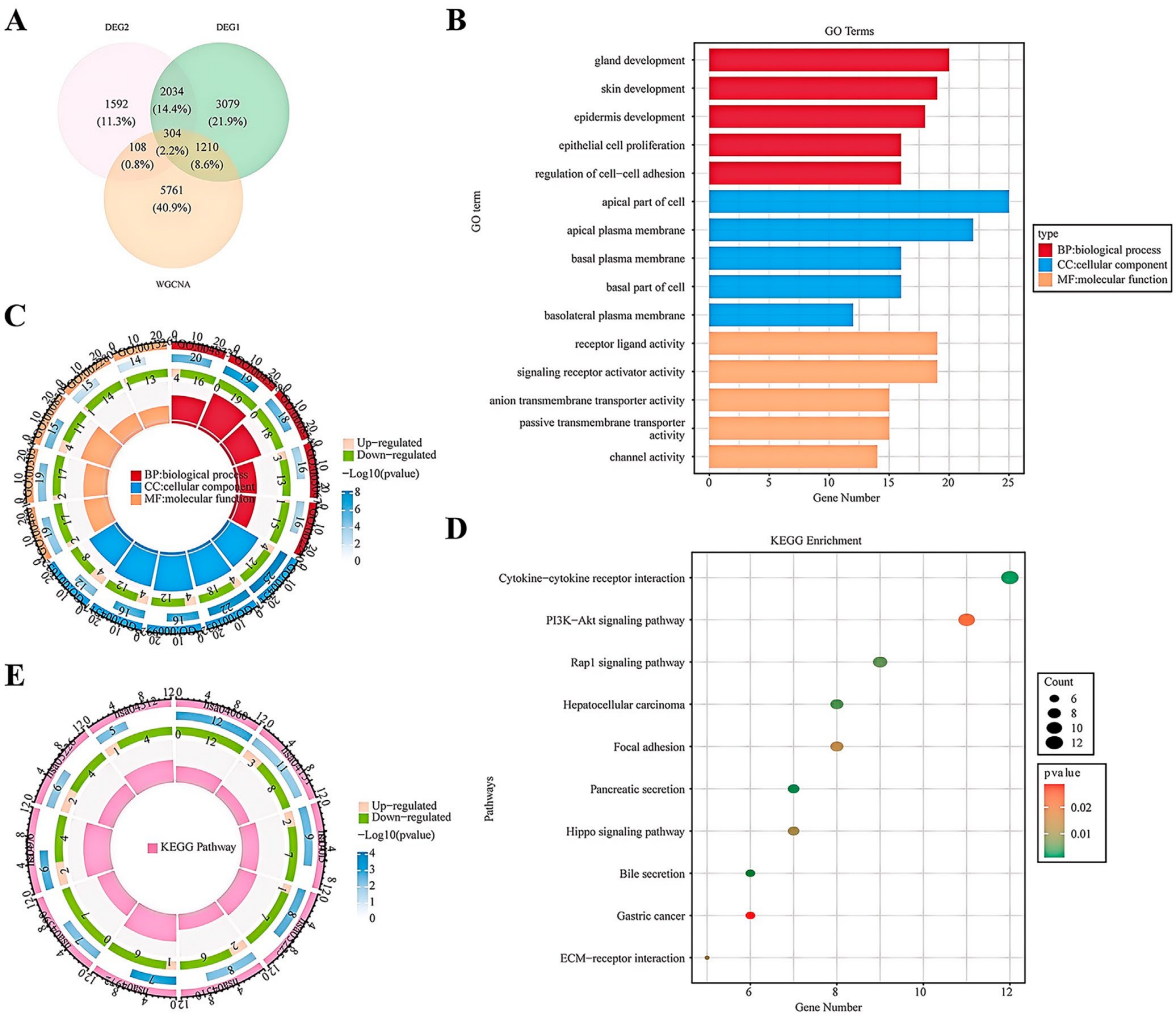
Identification and functional enrichment of candidate genes. (A) Venn diagram showing the intersection of DEGs1, DEGs2, and key WGCNA module genes, yielding 304 candidate genes. (B) Bar plot of the top 5 enriched Gene Ontology (GO) terms in the biological process (BP), cellular component (CC), and molecular function (MF) categories. (C) Dot plot of enriched GO terms, with color representing adjusted *p*‐value and dot size representing gene count. (D) Bar plot of the top 10 enriched KEGG pathways. (E) Dot plot of enriched KEGG pathways.

By intersecting DEGs1, DEGs2 and key module genes, 304 candidate genes were obtained (Figure 3A), which participated in 595 GO functional items, such as gland development, apical part of cell, receptor ligand activity (Figure 3B,C). Moreover, the selected genes were associated with 28 KEGG biological pathways, including the cytokine‐cytokine receptor interaction, phosphoinositide 3‐kinase (PI3K) signaling and so on (Figure 3D,E).

### 3.4 A Total of Six Prognostic Genes Were Screened

**FIGURE 4:**
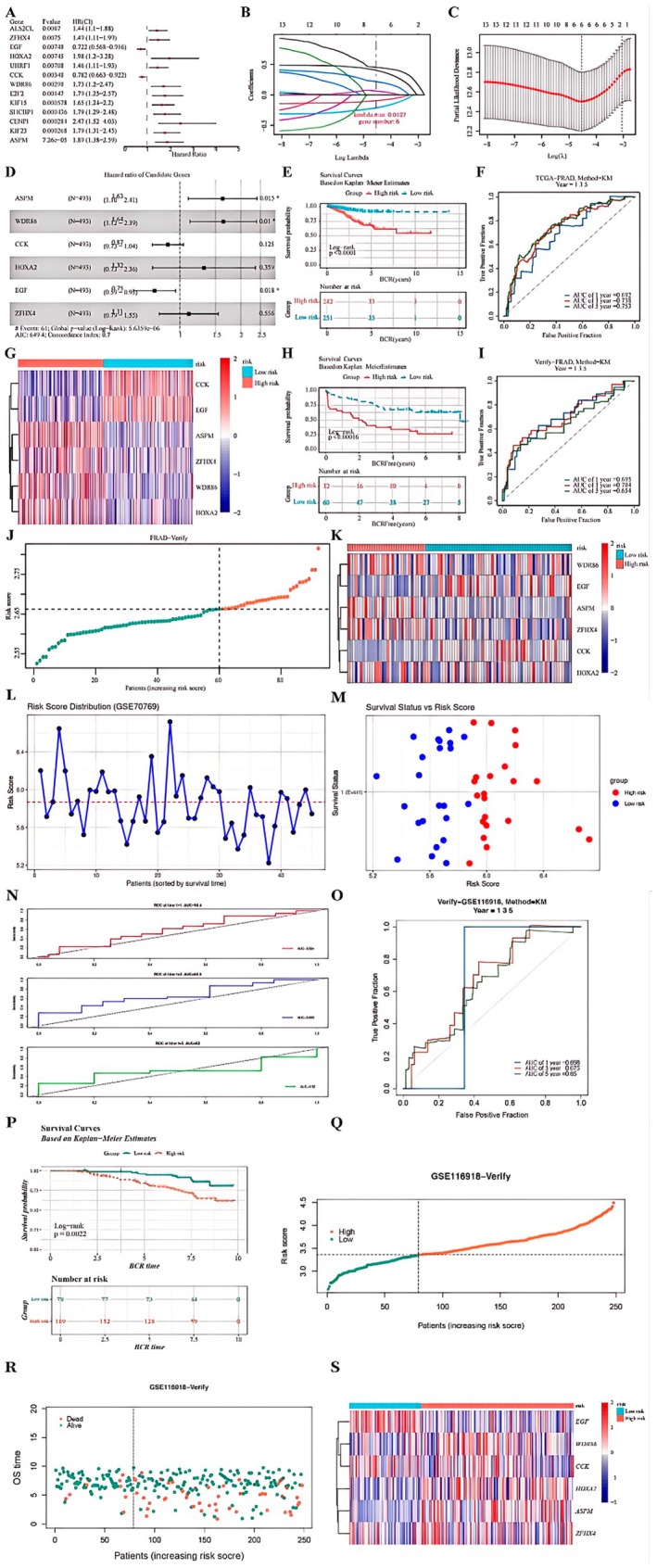
Construction and validation of a six‐gene prognostic signature. (A) Forest plot of 13 genes notably linked to OS within univariate Cox regression (*p* < 0.05). Risk ratios accompanied by 95% confidence bounds are shown. (B) Least absolute shrinkage and selection operator parameter trajectories for the 13 genetic loci. (C) Cross‐validation error plot for LASSO regression. The optimal λ (log scale) is indicated by the dotted line. (D) LASSO coefficient values for the six final prognostic genes. (E) Kaplan–Meier time‐to‐event plots for elevated‐and reduced‐risk categories within the TCGA model development set. (F) Time‐dependent ROC curves at 1, 3, and 5 years in the TCGA cohort. AUC values are shown. (G) Risk score distribution (top), survival status (middle), and expression heatmap of the six genes (bottom) in TCGA patients. (H) Kaplan–Meier curves for the GEO validation cohort (GSE70769). (I) Time‐dependent ROC curves in the GEO cohort. (J) Hazard index dispersion along with vital condition within the GEO patient set (K) Transcriptional intensity map of the six genes across GEO set. (L) Risk score distribution in the GSE70769 cohort sorted by survival time, with the red dashed line indicating the median risk score cutoff (range: 5.2–6.6). (M) Scatter plot of risk score versus biochemical recurrence status in the GSE70769 cohort. Red dots indicate high‐risk patients; blue dots indicate low‐risk patients. Higher risk scores are associated with greater probability of biochemical recurrence. (N) Time‐dependent ROC curves for biochemical recurrence‐free survival prediction in the GSE70769 cohort at 1 year (AUC = 0.584), 3 years (AUC = 0.666), and 5 years (AUC = 0.520). (O) Time‐dependent ROC curves of the six‐gene signature in GSE116918 at 1, 3, and 5 years. AUC values are indicated. (P) Kaplan–Meier overall survival curves for high‐ vs. low‐risk patients in GSE116918. *p* value from log‐rank test. (Q) Survival status distribution in GSE116918. (R) Risk score distribution of patients in GSE116918 (sorted by increasing risk score) (S) Expression heatmap of the six prognostic genes in GSE116918.

After the univariate COX regression analysis, a total of 13 genes were found (Figure 4A). Subsequently, ASPM, WDR86, CCK, HOXA2, EGF, and ZFHX4 were regarded as prognostic genes by LASSO‐COX analysis (Figure 4B–D). The risk model was calculated in TCGA‐PRAD (RiskScore = ASPM × (0.487) + WDR86 × (0.495) + CCK × (−0.138) + HOXA2 × (0.274) + EGF×(−0.29) + ZFHX4 × (0.101)), where PCa patients were categorized high‐ and low‐risk categories. Moreover, Kaplan–Meier survival curves then reflected the significant differences in survival between the stratified cohorts (Figure 4E). The prognostic performance of the risk model was further evaluated using time‐dependent ROC analysis. In the TCGA training cohort, the model achieved AUC values of 0.692, 0.738, and 0.753 at 1, 3, and 5 years, respectively (Figure 4F). To assess potential optimism in the training‐set estimates, internal validation was performed using 10‐fold cross‐validation, yielding a bias‐corrected 3‐year AUC of 0.733, which was consistent with the apparent AUC (0.738), confirming that the model performance was stable and not subject to meaningful overfitting. Later, mortality in PCa patients also increased as the risk score increased (Figure 4G). Furthermore. The risk score was further confirmed in GSE70769 by the Kaplan–Meier (K‐M), ROC curve and risk curve (Figure 4H–K). To extend validation beyond overall survival and provide a more comprehensive assessment of model utility, biochemical recurrence‐free survival (BCR‐FS) was additionally evaluated in the GSE70769 cohort using the same locked risk formula. Risk score distribution analysis showed that scores ranged from approximately 5.2–6.6, with patients above the median threshold classified as high‐risk (Figure 4L). Recurrence events were more concentrated in the high‐risk group, with higher overall risk scores relative to the low‐risk group, indicating a positive association between risk score and biochemical recurrence probability (Figure 4M). Time‐dependent ROC analysis yielded AUC values of 0.584, 0.666, and 0.520 at 1, 3, and 5 years, respectively, with the 3‐year time point demonstrating the best discriminative performance (Figure 4N). Taken together, the consistent directional concordance across two independent clinical endpoints within the GSE70769 cohort further supports the robustness of the six‐gene prognostic signature. In GSE116918, the signature yielded modest but significant prognostic discrimination (log‐rank *p* < 0.05), with time‐dependent AUC values showing consistent performance across 1, 3, and 5 years. Risk score, survival status, and gene expression heatmap trends were directionally consistent with earlier cohorts (Figure 4O–4S).

### 3.5 Predictive Accuracy of the Nomogram for PCa Patients

**FIGURE 5:**
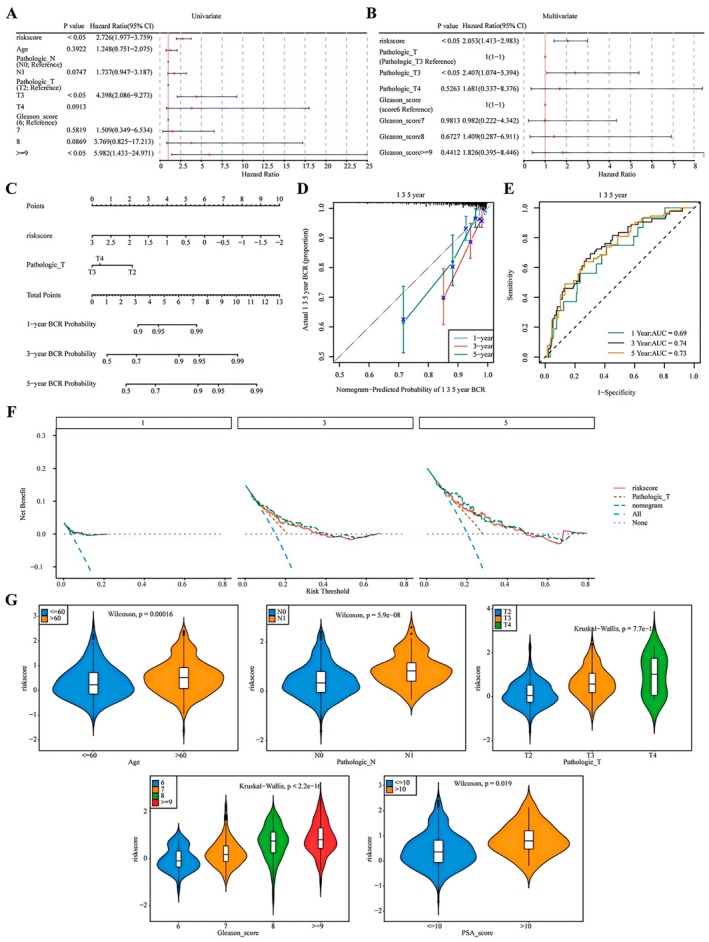
Independent prognostic analysis and nomogram construction. (A) Univariate Cox regression forest plot for clinical factors and risk score. (B) Multivariate Cox regression forest plot identifying independent prognostic factors. (C) Nomogram integrating risk score, pathological T stage, and Gleason score for predicting 1‐, 3‐, and 5‐year overall survival. (D) Calibration curves of the nomogram for 1‐, 3‐, and 5‐year survival probabilities. Perfect prediction is indicated by the diagonal dotted line. (E) Time‐dependent AUC curves comparing the nomogram, risk score, and individual clinical factors. (F) Decision curve analysis (DCA) showing the net benefit of the nomogram, risk score, and clinical factors. (G) Box plots of risk score stratified by different clinical characteristics (T stage, N stage, Gleason score, PSA). Wilcoxon test *p*‐values are indicated.

After formal assessment of the proportional hazards assumption using Schoenfeld residuals, PSA was found to significantly violate the PH assumption (*p* < 0.05) and was therefore excluded from the multivariable Cox model. All other variables, including the risk score, pathological T stage, N stage, Gleason score, and age, satisfied the PH assumption (*p* > 0.05) and were eligible for inclusion. Subsequently, the risk score, pathological T stage, and Gleason score were observed as linked with individual's survival (Figure 5A). Furthermore, multivariate Cox proportional hazards regression revealed risk score and pathological T stage as independent prognostic factors for PCa patients (Figure 5B). And their corresponding nomogram was also constructed (Figure 5C), which the area under time‐dependent AUC values surpassed 0.6 in all cases (Figure 5E). Additionally, the calibration curve showed no significant deviation in the accuracy of predicting 1/3/5‐year survival (Figure 5D). In terms of treatment efficacy, the model showed the highest treatment benefit (Figure 5F). These results collectively confirmed the accuracy and effectiveness of our nomogram prediction model. Eventually, risk scores were significantly different across these clinical indicators (Figure 5G).

### 3.6 Biological Functions Between Different Risk Groups Were Explored

**FIGURE 6:**
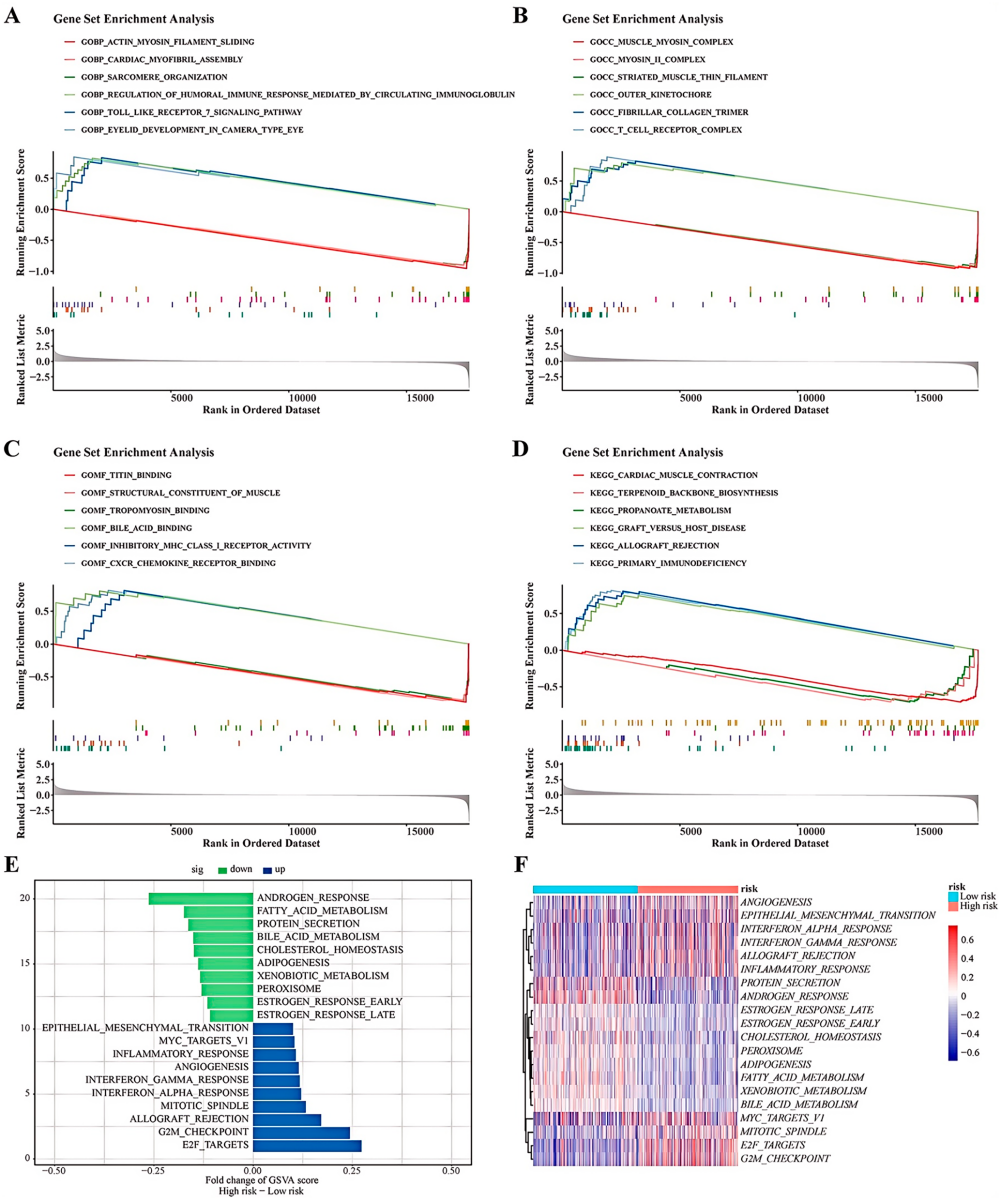
Functional annotation and pathway differences between risk groups. (A–C) Gene set enrichment analysis (GSEA) based on GO categories: (A) biological process (e.g., actin myosin filament sliding), (B) cellular component (e.g., muscle myosin complex), (C) molecular function (e.g., structural constituent of muscle). (D) GSEA based on KEGG pathways (e.g., cardiac muscle contraction). (E) Selected hallmark pathways showing significant differences: Androgen response, fatty acid metabolism (downregulated in high‐risk), E2F targets, G2M checkpoint (upregulated in high‐risk). Heatmap of GSVA scores for signature routes between elevated‐risk versus reduced‐risk categories.

In the GSEA, based on the GO background gene set, biological pathways involving genes from different risk groups included actin myosin filament sliding, cardiac myofibril assembly, etc. (Figure 6A). The cellular components involved comprised muscle myosin complex, myosin II complex, etc. (Figure 6B). Moreover, the molecular functions included structural constituent of muscle, tropomyosin binding, and so on (Figure 6C). Additionally, based on the KEGG background gene set, the genes are primarily enriched in biological pathways including cardiac muscle contraction, terpenoid backbone biosynthesis, etc. (Figure 6D). Subsequently, differential Hallmark pathways between risk groups were obtained; for instance, androgen response, fatty acid metabolism, etc. were inhibited, while E2F targets, G2M checkpoint, etc. were activated (Figure 6E,F).

### 3.7 Differences in 23 Immune Cells and 11 Immune Checkpoints Were Observed

**FIGURE 7:**
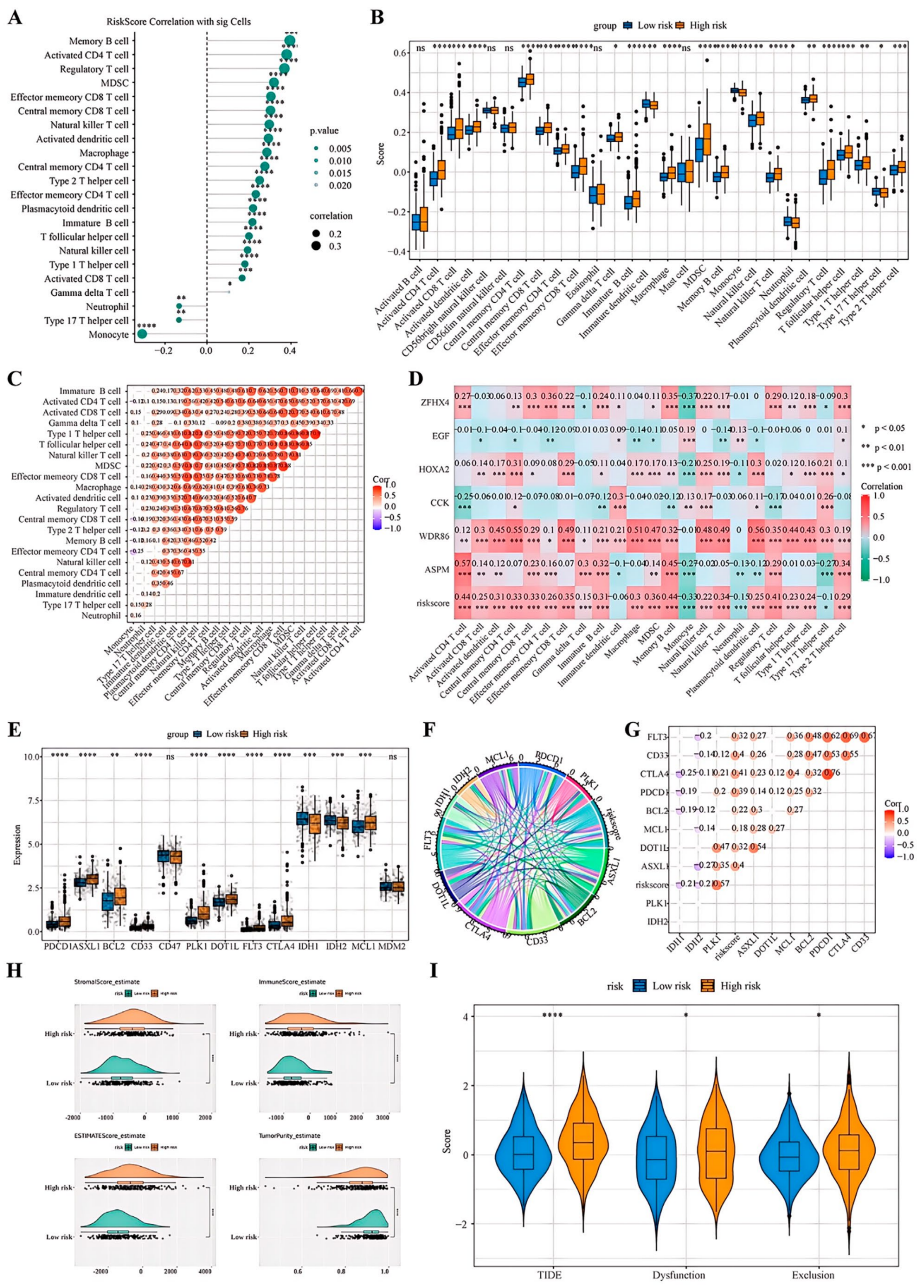
Characterization of the tumor immune microenvironment and immunotherapy response. (A) Risk score correlation with differential immune cells. (B) ssGSEA‐based immune infiltration analysis: Boxplot showing the differential expression of 28 immune cell types between high‐ and low‐risk groups; Heatmap showing the correlations among differential immune cell populations. (D) Correlation heatmap of biomarkers and differential immune cells. (E) Expression differences of immune checkpoints between high‐ and low‐risk groups. (F, G) Correlation among risk score, biomarkers, and immune checkpoints. (H) Comparison of ImmuneScore, StromalScore, tumor purity, and ESTIMATEScore among elevated‐ and reduced‐risk cohorts according to the ESTIMATE methodology. (I) Contrast of TIDE values between high‐ and low‐risk groups.

In this study, we quantified the enrichment scores of 28 immune cell subsets, including 23 immune cell subsets, particularly active CD4 T cell, active CD8 T cell, etc., demonstrated significant disparities across risk groups (Figure 7A,B). The correlation analysis demonstrated that the Type 1 T helper cell and T follicular helper cell exhibited highest correlation (cor = 0.9, *p* value < 0.05) (Figure 7C). Additionally, ZFHX4 showed the maximum negative association with Monocyte (cor = −0.37, *p* value < 0.05), ASPM showed the highest positive relationship with active CD4 T cell (cor = 0.44, *p* value < 0.05; Figure S1). Moreover, the risk score demonstrated the greatest positive correlation with Memory B cell (cor = 0.44, *p* value < 0.05) (Figure 7E). Furthermore, 11 immune checkpoints including PDCD1, ASXL1, BCL2, etc., showed significant differences between risk groups (Figure 7F,G), with the highest positive correlation observed between CTLA4 and PDCD1 (cor = 0.78). Besides, the ESTIMATE results exhibited significantly higher levels in the high‐risk group, with significant disparities observed in immune, stromal, and tumor purity across risk groups (Figure 7H). TIDE scores were significantly elevated within the elevated‐risk cohort, suggesting a computationally predicted propensity for immune evasion, though this inference requires validation in immunotherapy‐treated cohorts (Figure 7I). It is important to note that an elevated TIDE score reflects transcriptomic features associated with immune dysfunction, and should not be interpreted as direct evidence of immunotherapy resistance.

### 3.8 Lapatinib and ATRA Were Used for Molecular Docking With Prognostic Genes

**FIGURE 8:**
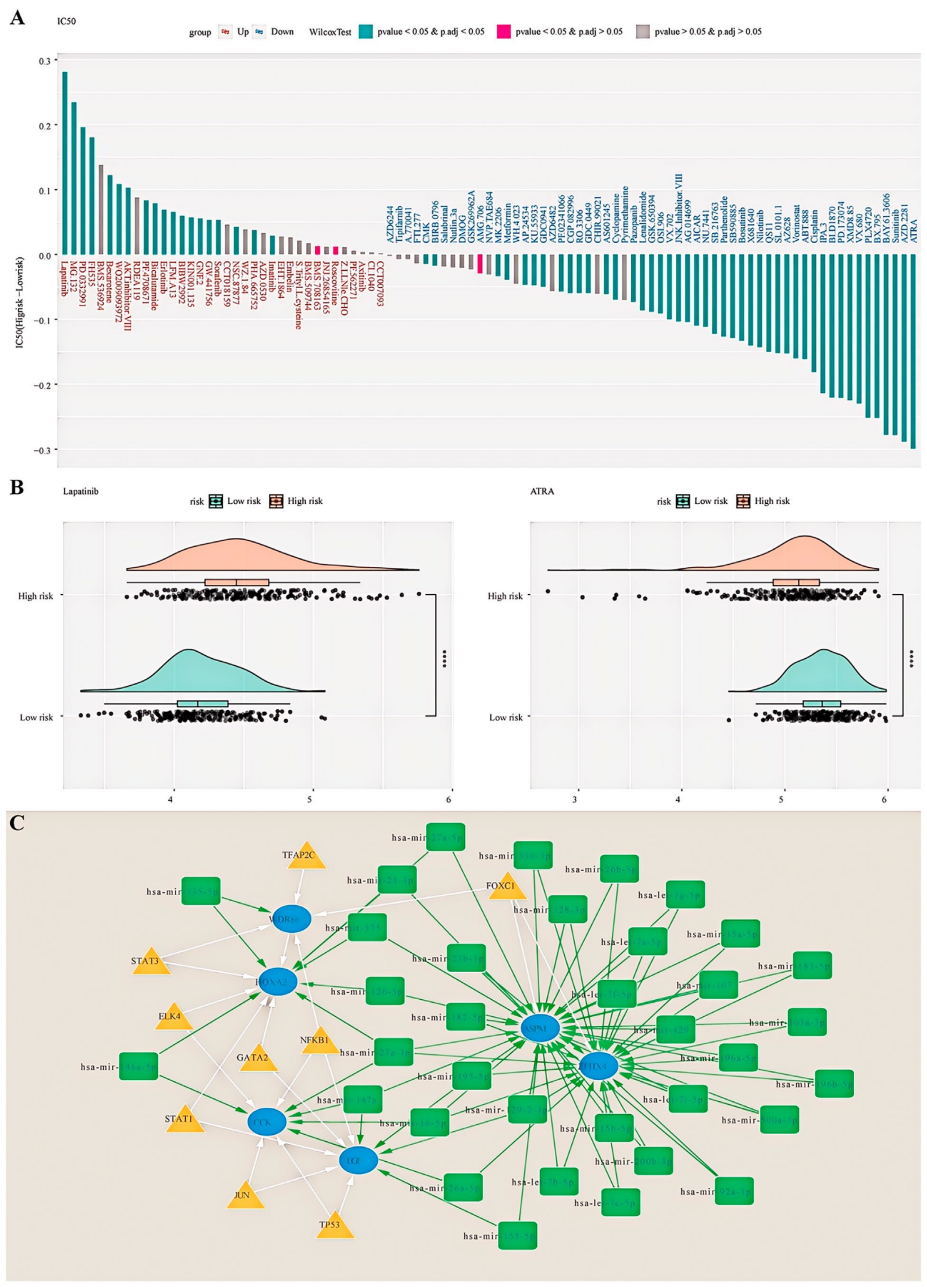
Drug sensitivity prediction and regulatory network. (A) Heatmap showing IC50 values of 67 drugs with significant differences between risk groups. (B) Box plots of predicted IC50 for lapatinib and all‐trans retinoic acid (ATRA) in high‐risk vs. low‐risk groups. (C) Regulatory network of miRNAs and transcription factors (TFs) targeting the six prognostic genes (e.g., hsa‐mir‐155‐5p‐EGF‐TP53).

In this study, significant differences were observed within the 50% inhibitory concentration estimates for 67 pharmaceutical agents across the hazard categories (Figure 8A), with the largest difference found between the Lapatinib and ATRA groups (Figure 8B). Lapatinib and ATRA were subjected to molecular docking. Both drugs demonstrated high binding affinity and stability with respect to prognostic genes, with Lapatinib binding energies ranging from −9.1 to −6.4 kcal/mol and ATRA ranging from −7.7 to −6.0 kcal/mol across the six targets (Table 1). After that, 35 miRNAs and 9 TFs were predicted to be corresponding to prognostic genes, and the regulatory relationships they formed in miRNA‐mRNA‐TF included hsa‐mir‐155‐5p‐EGF‐TP53, hsa‐mir‐147a‐JUN‐CCK, and so on (Figure 8C).

**TABLE 1:** Molecular docking results of lapatinib and ATRA with the six prognostic gene products.

TCMSP_ID	Common.name	Uniprot.ID	Autodock vina score (kcal/mol)
Lapatinib	ASPM	AF‐Q4G1H2	−9.1
Lapatinib	WDR86	AF‐A0A0C4DGX6	−7.0
Lapatinib	CCK	AF‐Q6FG82	−6.4
Lapatinib	HOXA2	AF‐O43364	−7.8
Lapatinib	EGF	AF‐L8EC91	−7.8
Lapatinib	ZFHX4	AF‐Q5U3C1	−7.0
ATRA	ASPM	AF‐Q4G1H2	−7.7
ATRA	WDR86	AF‐A0A0C4DGX6	−6.0
ATRA	CCK	AF‐Q6FG82	−6.0
ATRA	HOXA2	AF‐O43364	−6.2
ATRA	EGF	AF‐L8EC91	−6.9
ATRA	ZFHX4	AF‐Q5U3C1	−6.3

### 3.9 The 3 Key Cells Differentiate Into Different States

**FIGURE 9:**
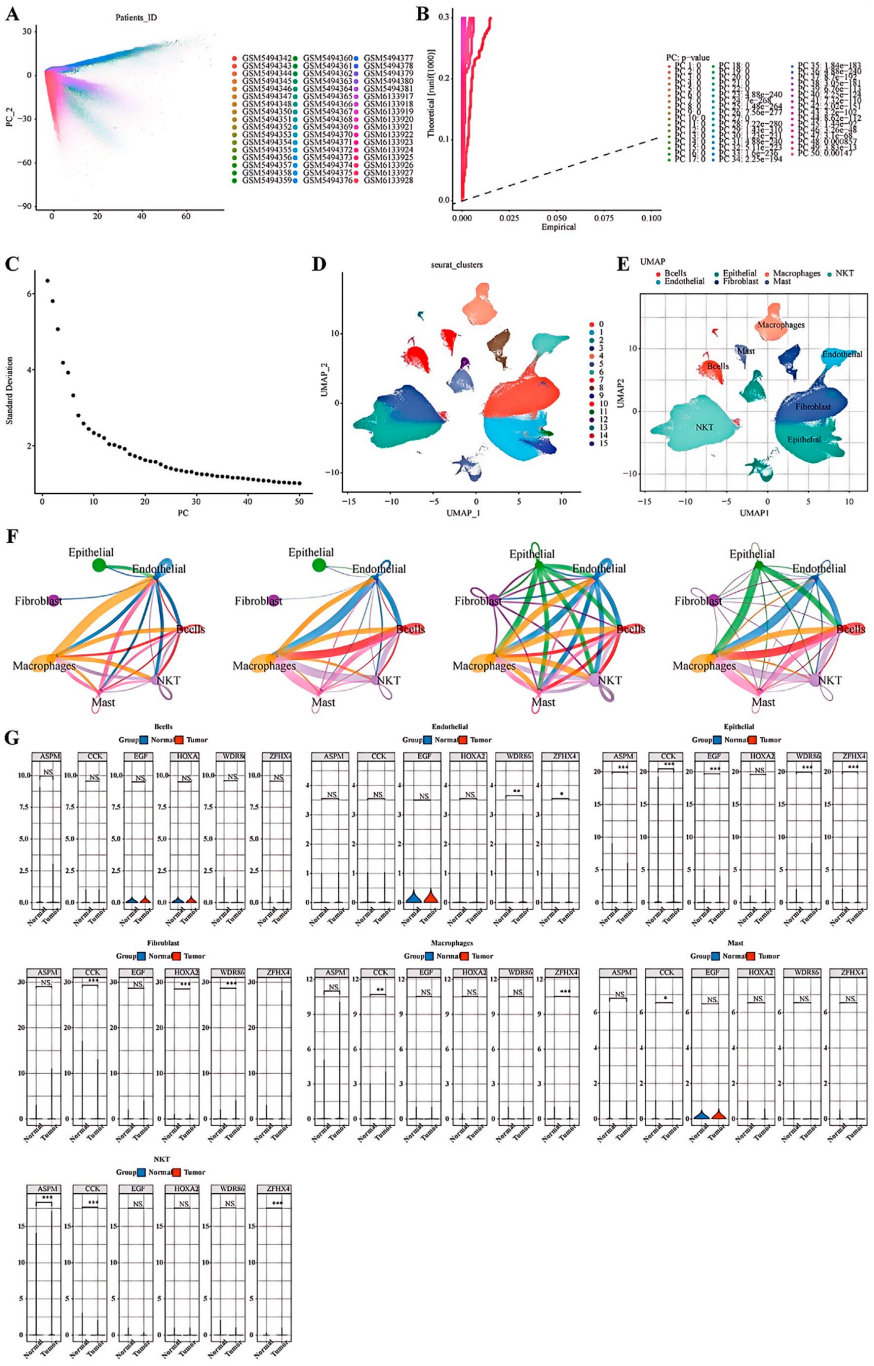
Single‐cell RNA‐seq analysis reveals cellular heterogeneity and expression of prognostic genes. (A) Quality control metrics: Number of genes, number of unique molecular identifiers (UMIs), and percentage of mitochondrial genes before and after filtering. (B) PCA plot of the top 2000 variable genes. (C) JackStraw plot to determine significant principal components. (D) UMAP plot showing dimensionality reduction and clustering of cell populations. (E) Plot showing cell type annotation. (F) CellChat analysis showing the number and strength of cell–cell communications in cancer vs. normal samples. (G) Point chart displaying the abundance of the half‐dozen predictive genetic loci among distinct cellular categories. Epithelial cells, fibroblasts, and NKT cells were identified as key expressors.

**FIGURE 10:**
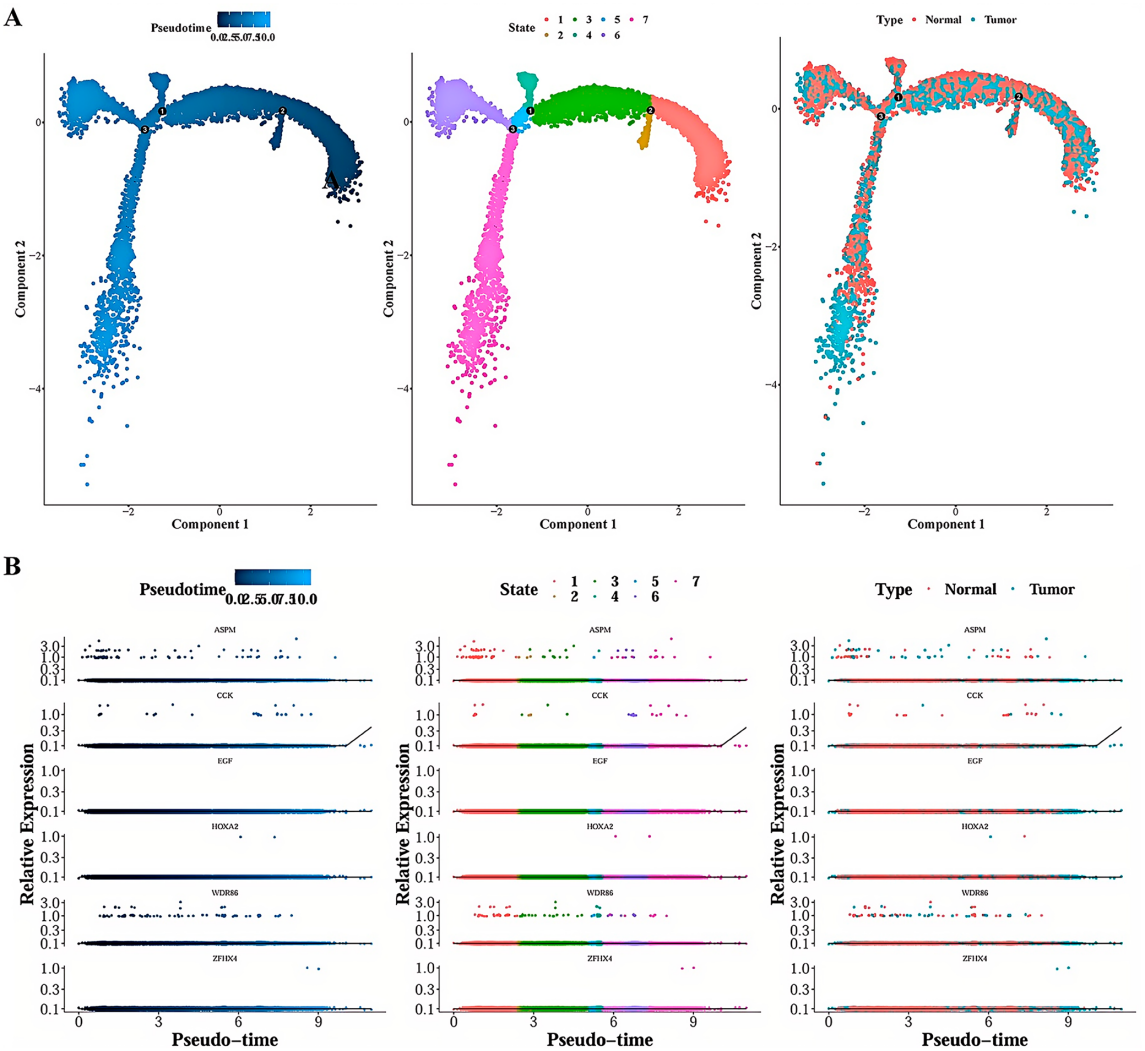
Pseudotime trajectory analysis of key cell populations. (A) Pseudotime trajectories of epithelial cells (left), fibroblasts (middle), and NKT cells (right), colored by pseudotime. (B) Differentiation states of each cell type: Epithelial cells (3 states), fibroblasts (3 states), NKT cells (6 states). Cells are projected onto UMAP and colored by state.

After filtering GSE181294, we selected the 2000 most variable genes for PCA analysis (Figure 9A–C). Top 50 PCs were clustered into 16 cell clusters (Figure 9D). A total of seven cell types were annotated from these cell clusters, including B cells, epithelial cells, macrophages, and others (Figure 9E). In cell communication analysis, the number of communications between endothelial cells significantly increased in the cancer group. In contrast, the number of cell communications between B cells, macrophages, and natural killer T (NKT) cells was higher in the normal group (Figure 9F). Pseudotemporal trajectory analysis revealed that epithelial cells, fibroblasts, and NKT cells differentiated into 3, 3, and 6 distinct states, respectively (Figure 10A,B). The number of states was determined by the number of trajectory branching points identified by Monocle2 in conjunction with unsupervised clustering consistency, rather than being predefined manually. Each state was biologically characterized based on dynamic gene expression patterns along the pseudotime axis, combined with cell cycle phase, functional phenotype, and transcriptional state differences. These state assignments were further cross‐referenced with known lineage markers to support the biological interpretation of distinct differentiation trajectories in the prostate cancer microenvironment.

### 3.10 Validation of Prognostic Gene Expression and Cross‐Platform Discrepancies

**FIGURE 11:**
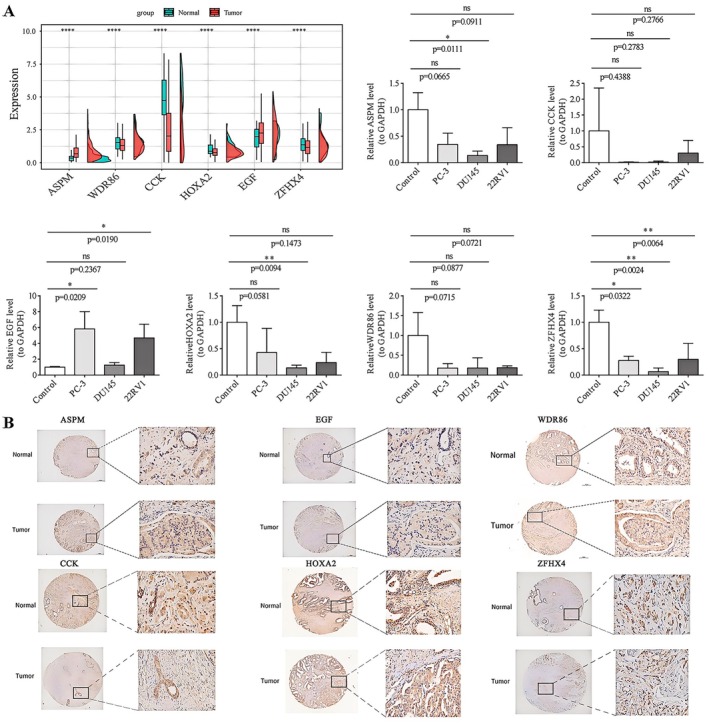
Laboratory confirmation of outcome‐predictive genetic transcription. (A) Box charts of the half‐dozen genetic loci's abundance within TCGA tumor tissues vs. adjacent normal tissues. qRT‐PCR in prostate cancer cell lines. Statistical significance was assessed by Wilcoxon test. (B) Immunohistochemistry (IHC) validation on a prostate cancer tissue microarray (TMA; PC‐1601; *n* = 64 patients, 192 cores, including paired tumor and adjacent normal tissues). Representative images (40×) of ASPM, CCK, EGF, HOXA2, WDR86, and ZFHX4 staining are shown. Statistical comparisons between cancer and adjacent normal tissues were performed using the Wilcoxon rank‐sum test.

In TCGA‐PRAD cohort, ASPM and EGF were significantly upregulated in tumor tissues, while WDR86, CCK, HOXA2, and ZFHX4 were downregulated (Figure 11A). qRT‐PCR in prostate cancer cell lines revealed both consistencies and discrepancies.

Consistent trends: ASPM was upregulated in DU145; HOXA2 downregulated in DU145; ZFHX4 downregulated in all three cell lines; EGF upregulated in PC‐3 and 22RV1 (*p* < 0.05).

Discrepant trends: WDR86 and CCK, downregulated in TCGA tumors, were upregulated in all cancer cell lines (PC‐3 and 22RV1 for WDR86; DU145 and 22RV1 for CCK; *p* < 0.05).

These discrepancies suggest that WDR86 and CCK expression may be influenced by tumor microenvironment components absent in cell lines, warranting further investigation using spatial transcriptomics or in vivo models.

### 3.11 Immunohistochemical Validation of Prognostic Gene Expression in Prostate Cancer Tissues

IHC validation on a tissue microarray confirmed protein‐level dysregulation of all six genes. ASPM was significantly upregulated in cancer tissues (median: 20.64 vs. 15.73 in adjacent normal, Wilcoxon *p* = 0.001), consistent with TCGA and qRT‐PCR (DU145). EGF showed elevated expression (64.42 vs. 58.41, *p* = 0.03), aligning with TCGA and qRT‐PCR (PC‐3, 22RV1). CCK was significantly upregulated (20.40 vs. 13.10, *p* = 0.01), contradicting TCGA mRNA data but matching qRT‐PCR (DU145, 22RV1). HOXA2 was downregulated (56.36 vs. 62.85, *p* = 0.037), consistent with TCGA and qRT‐PCR (DU145). WDR86 showed lower expression (64.00 vs. 70.16, *p* = 0.049). The trend was consistent with the TCGA data. However, this result contradicted the findings from the qRT‐PCR analysis in PC‐3 and 22RV1 cells. ZFHX4 was significantly downregulated (25.93 vs. 30.91, *p* = 0.015; Figure 11B). The results are consistently observed across all platforms.

In summary, ASPM, EGF, and CCK were upregulated, HOXA2, WDR86, and ZFHX4 were downregulated in cancer tissues. Cross‐platform discrepancies were observed (CCK: IHC vs. TCGA; WDR86: IHC vs. cell lines). These findings highlight the influence of post‐transcriptional regulation and the tumor microenvironment, requiring further multi‐level validation.

### 3.12 Promoter Methylation Profiling of the Six Prognostic Genes in Prostate Cancer

**FIGURE 12:**
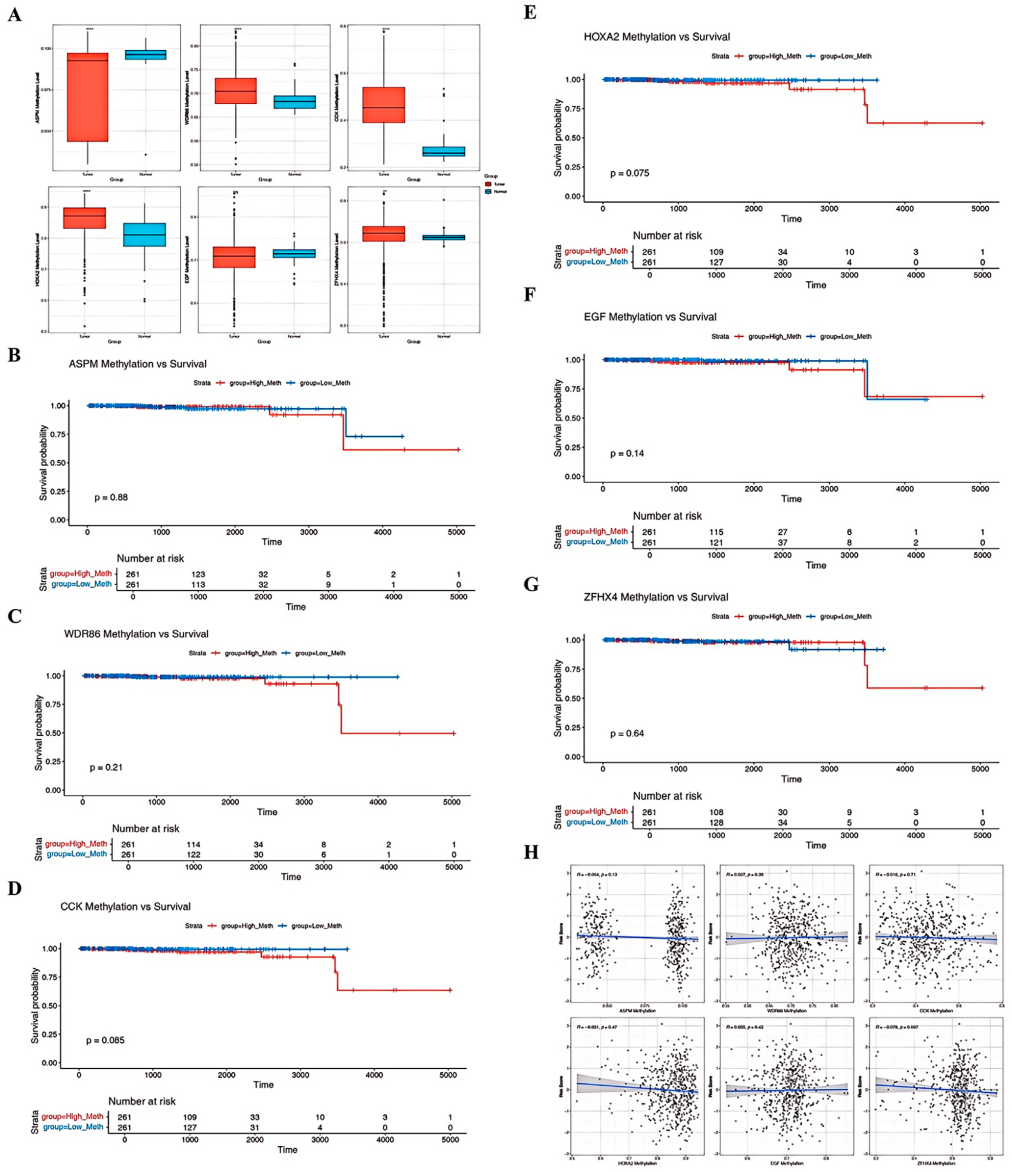
Promoter‐region DNA methylation analysis of the six prognostic genes in TCGA‐PRAD. (A) Box plots comparing promoter methylation beta values between tumor and adjacent normal tissues for ASPM, WDR86, CCK, HOXA2, EGF, and ZFHX4. Wilcoxon rank‐sum test; **p < 0.01, ****p < 0.001, ns: Not significant. (B–G) Kaplan–Meier survival curves stratified by median promoter methylation level (High_Meth vs. Low_Meth). (H) Scatter plots showing Pearson correlation between promoter methylation beta values and composite risk scores for each gene. *R* and *p* values are indicated.

To directly assess the epigenetic basis of the six‐gene signature, promoter‐region DNA methylation was analyzed using TCGA‐PRAD 450 K/850 K array data. WDR86, CCK, HOXA2, and ZFHX4 showed significantly elevated promoter methylation in tumor tissues relative to adjacent normal tissues (WDR86, CCK, HOXA2: *p* < 0.001; ZFHX4: *p* < 0.01), whereas ASPM was significantly hypomethylated in tumors (*p* < 0.001), consistent with its transcriptional upregulation and oncogenic role. EGF showed no significant methylation difference (*p* > 0.05; Figure 12A). Kaplan–Meier analysis showed that high CCK and HOXA2 methylation trended toward inferior overall survival, approaching but not reaching statistical significance (*p* = 0.085 and *p* = 0.075, respectively), while the remaining four genes showed no significant survival differences (Figure 12B–G). Correlation analysis between promoter methylation and the composite risk score revealed weak associations across all genes, with ZFHX4 showing the strongest marginal trend (*R* = −0.078, *p* = 0.067; Figure 12H). These findings confirm gene‐specific epigenetic dysregulation across the signature and provide direct supporting evidence for the methylation‐regulatory dimension of the proposed integrative framework.

## 4 Discussion

PCa remains a primary factor in cancer‐related mortality worldwide (Hao et al. 2024). It's known that epigenetic dysregulation and altered metabolism may lead to this result. Some literature has highlighted the crosstalk between DNA methylation and amino acid metabolism in cancer progression. However, an integrated prognostic signature remains poorly defined (Xu et al. 2023; Sun et al. 2022). In this study, we systematically integrated transcriptomic, epigenetic, and metabolic analyses to identify a novel six‐gene signature. The key biomarkers include ASPM, WDR86, CCK, HOXA2, EGF, ZFHX4. Hub genes are associated with methylation and amino acid metabolism. In this article, we also established its prognostic value through multi‐omics validation. Our immunohistochemical validation revealed both consistencies and cross‐platform discrepancies. This research provides insights into the complex biology of key genes and their relationship with the tumor microenvironment.

The key genes exhibit strong associations with PCa pathogenesis. However, their expression patterns across different platforms exhibited specific details. In our study, ASPM was upregulated across TCGA data, qRT‐PCR in DU145 cell lines, and IHC protein analysis (median: 20.64 vs. 15.73 in adjacent normal, *p* = 0.001). This multi‐platform trend supports ASPM's oncogenic role. Some research reveals that ASPM could maintain prostate cancer stem cell (CSC) populations though Wnt‐Dvl‐3‐β‐catenin signaling (Pai et al. 2019). In our investigation, IHC examination uncovered discordant ASPM expression within the prostate tissue. This expression pattern aligns with ASPM's CSC role. ASPM co‐localized with the CSC marker aldehyde dehydrogenase (ALDH) in tissues. The expression of ASPM correlated inversely with patient relapse‐free survival. It also maintains the cancer stem cell population by enhancing Wnt‐β‐catenin signaling. The downregulation reduces ALDH+ stem cells, and suppress tumor invasion and metastasis (Pai et al. 2019; Chan et al. 2024). EGF demonstrated consistent upregulation across all platforms (TCGA, qRT‐PCR in PC‐3/22RV1, and IHC with median 64.42 vs. 58.41, *p* = 0.03). This aligns with EGF's role in castration‐resistant PCa progression. It may contribute to PCa to avoid the growth restrictions from androgen deprivation. This biological behavior requires activation of G0/G1‐to‐S transition via p38 mitogen‐activated protein kinase (MAPK) signaling pathway (Rodríguez‐Berriguete et al. 2016). The consistent upregulation of EGF across both tissue and cell line models suggests that its oncogenic role may be relatively robust across different cellular contexts, though formal microenvironmental dependency studies are needed to confirm this. HOXA2 and ZFHX4 demonstrated downregulation across TCGA and IHC platforms. HOXA2 showed downregulation in DU145 cells. HOXA2 suppresses tumors by modulating integrins to constrain metastasis (Deneyer et al. 2019). ZFHX4's consistent downregulation is consistent with a tumor‐suppressive role, potentially mediated in part through transcriptional regulation of epithelial differentiation genes, though mechanistic validation is required.

In this study, cross‐platform discrepancies in CCK and WDR86 uncovered microenvironment‐cell interactions. CCK demonstrated upregulation at the protein level in cancer tissues by IHC (median 20.40 vs. 13.10, *p* = 0.01) and in PCa cell lines (DU145 and 22RV1). But, it showed downregulation in TCGA data. This pattern may be explained by complementary mechanisms. First, neuroendocrine differentiation may represent a plausible explanation. CCK (cholecystokinin) is a neuroendocrine peptide, and its receptor (CCK‐B/gastrin receptor) has been documented in various human tumors (Smith et al. 2016). Focal neuroendocrine differentiation is a well‐recognized phenomenon in prostate cancer. In bulk RNA‐seq analysis, the signal from scattered neuroendocrine cells may be diluted by the predominant adenocarcinoma cell population. However, we acknowledge that this hypothesis was not directly tested in the current study. Stratified analysis of TCGA‐PRAD samples by established neuroendocrine differentiation scores (e.g., neuroendocrine prostate cancer (NEPC) scores derived from Beltran et al.) or re‐examination of CCK expression in neuroendocrine marker‐positive versus ‐negative tumor regions would be required to substantiate this interpretation. Such analyses are planned for future work. Notably, our regulatory network analysis identified hsa‐mir‐147a as a predicted upstream regulator of CCK, providing an internally consistent candidate mechanism for post‐transcriptional suppression of CCK mRNA despite protein‐level accumulation, though direct experimental validation remains to be performed. The elevated CCK expression in DU145 and 22RV1 cells supports this interpretation. Second, post‐transcriptional regulation represents another plausible but unvalidated mechanism. Discordance between mRNA and protein levels is a well‐documented phenomenon in cancer biology, mediated by microRNAs, RNA‐binding proteins, and differential protein stability (Koussounadis et al. 2015). Nonetheless, we did not perform miRNA‐target validation or RNA‐binding protein interaction assays for CCK in this study; this explanation therefore remains speculative. WDR86 showed a distinct cross‐platform pattern: consistent downregulation across two independent tissue‐level datasets (TCGA bulk RNA‐seq and IHC protein quantification, *n* = 64 patients, median H‐Score 64.00 vs. 70.16, *p* = 0.049), contrasting with upregulation observed in isolated cancer cell lines (PC‐3 and 22RV1). We acknowledge that formal cross‐platform heterogeneity testing (e.g., Cochran's *Q* test or *I* ^2^ statistic) was not performed. These methods require pooling of comparable effect sizes with known variance from independent studies; however, the three platforms employed here (bulk RNA‐seq, IHC protein quantification, and cell line qRT‐PCR) measure fundamentally different molecular quantities with incomparable variance structures, precluding valid statistical pooling under a meta‐analytic framework. Instead, the directional concordance between TCGA and IHC—two independent tissue‐level measurements—provides qualitative evidence that WDR86 downregulation represents a consistent in vivo phenomenon. The divergence in cell lines most plausibly reflects the absence of microenvironmental suppressive signals in vitro, such as stromal‐derived factors, immune cell interactions, or epigenetic cues that may dominate WDR86 regulation in the native tumor context. It should be noted that this mechanistic interpretation remains a hypothesis, as no direct experimental evidence—such as conditioned medium transfer or stromal co‐culture assays—was generated in the current study. Validation through spatial transcriptomics or in vivo models is warranted.

Functional annotation was performed utilizing GSVA. In high‐risk patients, androgen response and fatty acid metabolism pathways were suppressed. E2F targets and G2M checkpoints were activated. In aggressive PCa patients, androgen receptor signaling loss is linked to neuroendocrine differentiation and disease progression. Downregulation of androgen response pathways is consistent with castration‐resistant development. It reflects tumor adaptation to escape androgen dependence (Senapati et al. 2023). The activation of E2F targets and G2M checkpoint pathways indicates cell‐cycle dysregulation. The dysregulation drives uncontrolled proliferation (Walston et al. 2021; Engeland 2022). Fatty acid metabolism suppression in high‐risk patients is striking. Altered lipid metabolism has been reported in PCa progression and therapy resistance (Currie et al. 2013; Jin et al. 2021). These alterations provide novel insights into the biological behavior of high‐risk tumors using our signature.

Immune correlation research exhibited relationships between candidate prognostic markers and immunological cell subsets. It uncovered TME‐methylation‐metabolism links. ZFHX4 exhibited the greatest negative correlation with monocyte infiltration (cor = −0.37, *p* < 0.05). Given the correlational nature of this analysis, it remains unclear whether ZFHX4 loss drives monocyte recruitment, or whether monocyte‐derived microenvironmental signals suppress ZFHX4 expression in tumor cells. Functional studies are needed to establish the directionality of this relationship. Monocytes and tumor‐associated macrophages play complex roles in PCa. It functions in both tumor‐promoting and anti‐tumor behaviors. The function may depend on their biological state and microenvironmental conditions (Boutilier and Elsawa 2021; Li et al. 2023). ASPM showed the strongest positive correlation with activated CD4^+^T cells (cor = 0.44, *p* < 0.05). This association may reflect a compensatory adaptive immune response to ASPM‐driven tumor proliferation; however, the reverse possibility—that an activated CD4^+^T cell‐enriched microenvironment selectively favors expansion of ASPM‐expressing tumor cells—cannot be excluded from correlational data alone. Whether these activated CD4^+^ T cells exert anti‐tumor effector function or undergo exhaustion and Treg differentiation in advanced PCa remains to be determined (Montauti et al. 2024). These elevated TIDE scores in the high‐risk cohort reflect transcriptomic patterns suggestive of a potential tendency toward immune evasion, rather than confirmed immune evasion capacity or resistance to immunotherapy. All these results suggested that based on computational TIDE scoring alone, high‐risk tumors showed a predicted propensity for immune evasion; however, whether this translates to differential responsiveness to immune checkpoint blockade remains to be validated in prospective immunotherapy cohorts, as TIDE has not been formally validated as a predictive biomarker in prostate cancer. Moreover, TIDE scores were significantly elevated in the high‐risk group. It should be noted that TIDE is a transcriptome‐based computational algorithm originally developed and validated in melanoma and other immune checkpoint inhibitor (ICI)‐responsive tumor types; its predictive value in prostate cancer—a tumor type with historically limited response to ICI monotherapy—has not been prospectively validated (Jiang et al. 2018; Sharma et al. 2021). Therefore, the elevated TIDE scores observed here should be interpreted as a hypothesis‐generating signal reflecting transcriptomic patterns associated with immune dysfunction, rather than a validated prediction of differential responsiveness to immune checkpoint blockade.

Single‐cell profiling revealed major cell subsets: epithelial cells, fibroblasts, and NKT cells. Prognostic gene‐expressing cells displayed distinct differentiation states utilizing pseudotemporal analysis. Malignant epithelial cells are the key drivers of PCa progression. The plasticity facilitated epithelial‐mesenchymal transition (EMT) and invasive phenotypes (Nanda et al. 2022; Castellón et al. 2022). Cancer‐associated fibroblasts remodel the extracellular matrix, secrete growth factors, and promote angiogenesis. All these biological functions lead to tumor progression and metastasis (Wang et al. 2024; Hu et al. 2023). We identified NKT cells as key expressors of prognostic genes. In some research, these innate‐like lymphocytes bridge innate and adaptive immunity, directly targeting PCa cells through natural killer group 2D (NKG2D) recognition (Bashiri Dezfouli et al. 2021; Braud et al. 2022). The reduced NKT cell communication observed in tumor samples is consistent with an immunosuppressive microenvironment, though whether this reduction causally contributes to immune evasion or poor outcomes requires functional validation. Pseudotemporal trajectories revealed multiple differentiation states for key cell populations. This confirmed the cellular heterogeneity within PCa. Prognostic genes may also drive specific differentiation states linked to aggressive disease.

Drug sensitivity analysis identified lapatinib and ATRA as key compounds. It suggested potential therapeutic vulnerabilities. Lapatinib, a small‐molecule dual tyrosine kinase inhibitor directed against human epidermal growth factor receptor 2 (HER2)/epidermal growth factor receptor (EGFR) (Zhu et al. 2023). In some research, lapatinib has shown efficacy across multiple cancers. It may be particularly relevant in AR‐negative PCa because these pathways are co‐opted for survival and proliferation (Martiniano 2021; Siatis et al. 2023; Maeda et al. 2022). ATRA (all‐trans retinoic acid) induces differentiation in various malignancies and restores retinoblastoma protein (RB)/E2F control in neuroendocrine PCa (Ma et al. 2022; Dong, Peng, et al. 2023). The differential sensitivity to these agents varies between risk groups. This suggests our key compounds could inform personalized therapeutic strategies. It may lead to guidance for drug repurposing in patient stratification. The molecular docking analysis predicted stable associations between these compounds and the prognostic gene products. This provides evidence of their potential direct or indirect interactions. All these need further experimental validation.

This study has several limitations that should be acknowledged. First, systematic cellular functional assays—including gene knockdown or overexpression experiments coupled with proliferation, migration, and invasion assays—were not performed for the six signature genes (ASPM, WDR86, CCK, HOXA2, EGF, ZFHX4) in this study. While multi‐platform expression validation through TCGA analysis, qRT‐PCR, and IHC provides consistent evidence for their dysregulation in PCa, direct experimental demonstration of their functional roles in cancer progression remains to be established. Such functional characterization is designated as a priority objective in our subsequent research program. Second, the clinical translation of the nomogram requires prospective cohorts to validate its predictive performance. Cross‐platform discrepancies were observed for CCK and WDR86. This highlights the need for spatial transcriptomics or in vivo models to discover the microenvironmental signals for hub genes. Third, the paradoxical expression patterns of CCK and WDR86 across platforms prompted three mechanistic hypotheses—neuroendocrine dilution in bulk sequencing, post‐transcriptional regulation, and microenvironmental suppression—none of which were experimentally validated in this study. Direct experimental validation—including neuroendocrine scoring of TCGA‐PRAD samples for CCK and stromal co‐culture assays for WDR86—is planned as a priority in subsequent studies. Future work incorporating neuroendocrine scoring of TCGA samples, spatial transcriptomics, and stromal co‐culture models is necessary to resolve these discrepancies and confirm the proposed mechanisms.

In conclusion, this study delineates a methylation‐amino acid metabolism axis in PCa. The biomarker genes include ASPM, WDR86, CCK, HOXA2, EGF, and ZFHX4, participating in clinical prognosis, immune modulation, and therapeutic targeting. The cross‐platform discrepancies observed in hub gene CCK and WDR86 highlight the importance of multi‐level validation. It suggests that these genes may serve as sensors of tumor microenvironment. CCK's protein‐level upregulation in the context of neuroendocrine differentiation. WDR86's microenvironment‐dependent suppression represent biologically meaningful patterns invisible to transcriptomic analysis alone. Our findings provide a translational framework for precision oncology. It may bridge epigenetic‐metabolic crosstalk to disease aggressiveness and offer potential biomarkers for risk‐stratified therapy and immunotherapy approaches in prostate cancer.

## 5 Conclusions

This study establishes a six‐gene signature (ASPM, WDR86, CCK, HOXA2, EGF, ZFHX4). The signature integrates DNA methylation and amino acid metabolism pathways in prostate cancer (PCa). It effectively stratifies patients by overall survival risk. This signature demonstrates robust predictive performance across independent cohorts. High‐risk tumors exhibit suppressed androgen response and fatty acid metabolism, alongside activated E2F/G2M checkpoint pathways.

The signature links with an immunosuppressive tumor microenvironment. It includes altered immune infiltration and elevated TIDE scores. Single‐cell analysis identifies epithelial cells, fibroblasts, and NKT cells as key expressors, with distinct differentiation trajectories in aggressive disease. Cross‐platform discrepancies for CCK and WDR86 underscore the influence of post‐transcriptional regulation and microenvironmental context.

In conclusion, this methylation‐metabolism signature predicts prognosis and reflects key biological features of aggressive PCa. These findings provide a framework for integrating epigenetic–metabolic crosstalk into precision oncology. The findings would support further investigation of these genes as biomarkers for risk stratification and therapeutic targeting.

## Author Contributions

**Chao Zhang:** data curation, investigation, writing – review and editing, resources, writing – original draft, validation. **Kai Wu:** conceptualization, formal analysis, writing – review and editing, funding acquisition, writing – original draft, validation. **Likun Liu:** conceptualization, formal analysis, writing – review and editing, writing – original draft.

## Funding

This work is supported by a Four “Batches” Innovation Project of Invigorating Medical through Science and Technology of Shanxi Province program grant (2023XM050 to Kai Wu), the Science and Education Cultivation Fund of the National Cancer and Regional Medical Center of Shanxi Provincial Cancer Hospital grant (QH2023044 to Kai Wu), Science and Technology of Shanxi Province program grant (2025ZYYB059 to Kai Wu).

## Ethics Statement

This study utilized publicly available transcriptomic data from The Cancer Genome Atlas (TCGA) and Gene Expression Omnibus (GEO) databases, for which ethical approval and informed consent were obtained in the original studies. The human prostate cancer cell lines (WPMY‐1, PC‐3, DU145, 22RV1) were commercially obtained from Procell Life Science & Technology Co. Ltd. (Wuhan, China), and their use does not require additional ethics approval. The tissue microarray (TMA; PC‐1601) containing paired tumor and adjacent normal specimens was commercially sourced and fully anonymized; therefore, no further institutional ethics approval or patient consent was required. This study conforms to the ethical standards of the relevant national and institutional committees. “Not applicable” is confirmed.

## Conflicts of Interest

The authors declare no conflicts of interest.

## Supporting information


**Figure S1:** Correlation heatmap of the six prognostic genes with immune cell subsets.

**Table S1:** Primer sequences.

**Table S2:** Differentially expressed genes between prostate cancer and adjacent normal tissues (DEGs1).

**Table S3:** Differentially expressed genes between high and low amino acid metabolism‐related gene (AAMRG) score groups (DEGs2).

## Data Availability

The datasets generated during and analysed during the current study are available from the corresponding author on reasonable request.
