# Supplementary figures and images for: An Integrative Methylation‐Metabolism Gene Signature Defines Prognosis and Immunosuppressive Microenvironment in Prostate Cancer
Source: Chem Biol Drug Des. 2026 Jul 20;108(1):e70360. doi: 10.1111/cbdd.70360 (PMC13386026; doi:10.1111/cbdd.70360)

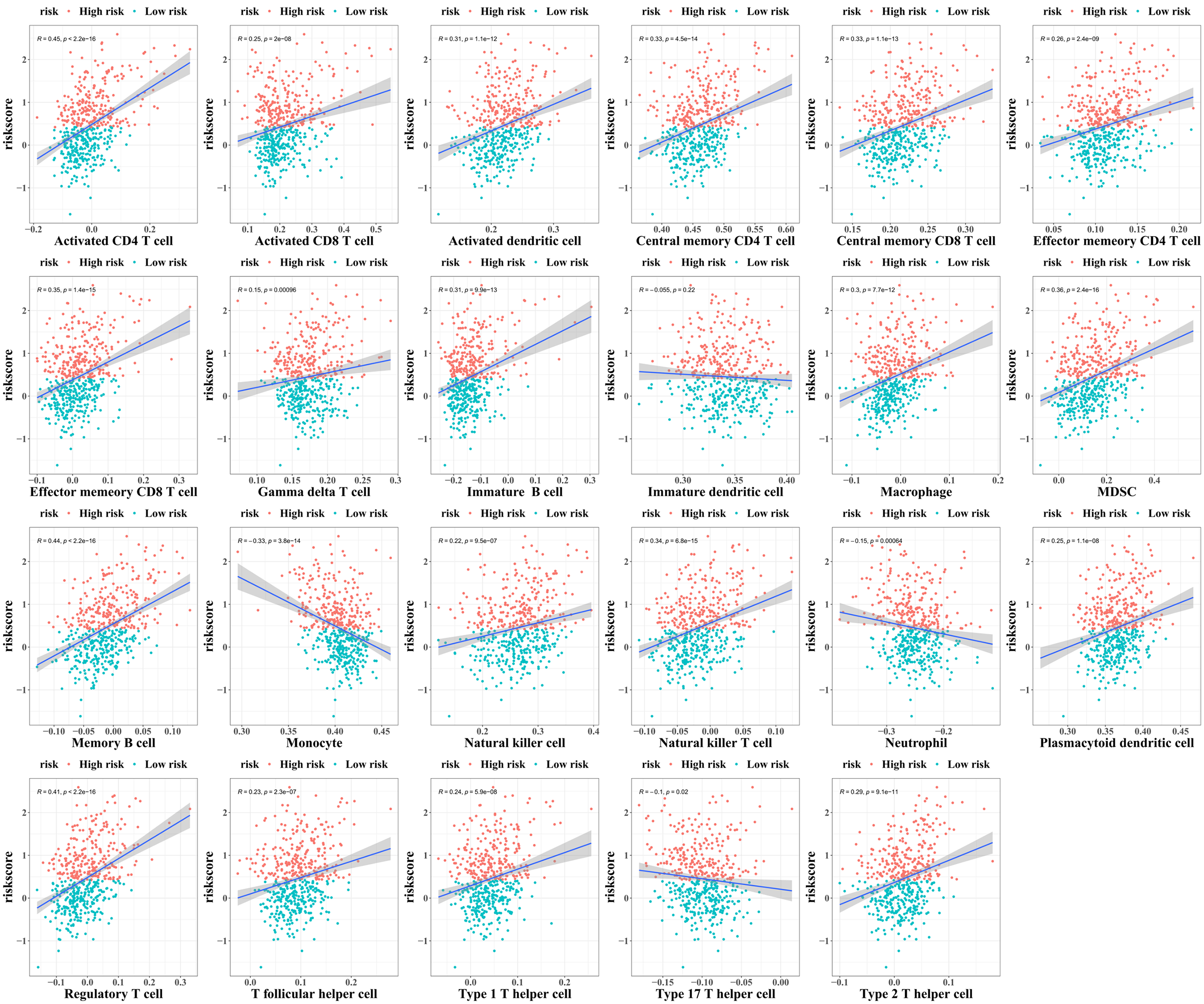

Supplement: Supplementary file 1 — Figure S1: Correlation heatmap of the six prognostic genes with immune cell subsets. [file CBDD-108-e70360-s004.png]
