# Supplementary material for: An Integrative Methylation‐Metabolism Gene Signature Defines Prognosis and Immunosuppressive Microenvironment in Prostate Cancer
Source: Chem Biol Drug Des. 2026 Jul 20;108(1):e70360. doi: 10.1111/cbdd.70360 (PMC13386026; doi:10.1111/cbdd.70360)
Supplement: Supplementary file 2 — Table S1: Primer sequences. [file CBDD-108-e70360-s003.docx]

****Table S1. Primer sequences****

| ****Primer**** | ****Sequence**** |  |  |
| --- | --- | --- | --- |
| ASPM F | TGCAGCACTCGTCATTCAGA |  |  |
| ASPM R | CTGAATTGTAACTGTAGCCCAAAGA |  |  |
| WDR86 F | CTTGAAAAGGACTGTCGGGC |  |  |
| WDR86 R | TGAGTGGTGTCGGGTGAAAG |  |  |
| CCK F | TTGCATCTAAGACGGGTGGG |  |  |
| CCK R | CCTTTGAGTTCGAGTCCGCT |  |  |
| HOXA2 F | AGAATCCCTGGAAATCGCCG |  |  |
| HOXA2 R | CCTCCGGTTCTGAAACCACA |  |  |
| EGF F | CCTGTGGGATGCAGCATGTA |  |  |
| EGF R | GTGGAGTAGAGTCAAGACAGTGAA |  |  |
| ZFHX4 F | TAATGAGCTGCCGCCTGAAA |  |  |
| ZFHX4 R | ACATGCACACTTAGGGCCTC |  |  |
| Internal reference R-GAPDH F | GACCCCTTCATTGACCTCAAC |  |  |
| Internal reference R-GAPDH R | GCCATCACGCCACAGCTTTCC |  |  |
|  |  |  |  |
